# Comparing the effects of computerized versus manual methods of identifying point-specific acupuncture as an adjunct to physiotherapy in the management of knee osteoarthritis: A randomized controlled trial protocol

**DOI:** 10.1371/journal.pone.0313761

**Published:** 2025-01-13

**Authors:** Lee Chai Li, Mohd Azzuan Ahmad, Tan Chee Hou, Angeline Low Ann Je, Lee Zi Lin, Tay Yan Ling, Peng Yan

**Affiliations:** 1 Physiotherapy Program, Centre for Rehabilitation and Special Needs Studies, Faculty of Health Sciences, Universiti Kebangsaan Malaysia, Kuala Lumpur, Malaysia; 2 Grand Care Rehab Seremban (Physiotherapy & Acupuncture), Seremban, Negeri Sembilan, Malaysia; Sheikh Hasina National Institute of Burn & Plastic Surgery, BANGLADESH

## Abstract

**Background:**

Knee osteoarthritis (KOA) is a prevalent condition causing significant pain and functional impairment. Acupuncture has shown promise as an adjunctive therapy, but conventional manual selection of acupoints lacks standardization. The Acugraph system provides a computerized method for identifying acupoints, potentially enhancing treatment precision.

**Objective:**

This study aims to compare the effects of computerized Acugraph-guided acupuncture versus manually selected acupuncture as adjuncts to physiotherapy in managing KOA.

**Methods:**

A randomized, double-blind controlled trial will be conducted with 50 participants diagnosed with mild to moderate KOA. Participants will be randomly assigned to one of two groups: Group 1 Comp-AcuPhysio (n = 25), receiving Acugraph-guided acupuncture with physiotherapy, or Group 2 Man-AcuPhysio (n = 25), receiving manually selected acupuncture with physiotherapy. Both groups will undergo 12 weekly treatment sessions, each lasting 60 minutes. Outcome measures, including the Knee Injury and Osteoarthritis Outcome Score, active knee flexion range, Timed Up and Go test, Visual Analog Scale for pain, Short Form-36 health survey, and Personal Integrated Energetics score, will be assessed at baseline and immediately post-intervention. An intention-to-treat analysis will be applied. Changes from baseline to 12 weeks will be analyzed using repeated measures analysis of variance for both within-group and between-group comparisons.

**Results:**

This study will provide a definitive assessment of the effectiveness of computerized Acugraph-guided acupuncture compared to manually selected acupuncture as supplementary treatments alongside KOA physiotherapeutic rehabilitation.

**Conclusion:**

This trial will offer insights into how incorporating technology-driven approaches, such as Acugraph, with physiotherapy can enhance the customization and effectiveness of KOA management, leading to improved clinical outcomes. These results could advocate for the integration of technological tools in acupuncture to boost treatment precision and efficacy for KOA.

**Trial registration:**

Australian New Zealand Clinical Trials Registry (ACTRN12624000646549p).

## Introduction

Knee osteoarthritis (KOA) is a multifaceted condition affecting various components of the knee joint, including calcified cartilage, subchondral bone, ligaments, and synovial fluid [1]. The persistent inflammatory response associated with KOA exacerbates impairments and imposes significant functional limitations [1, 2]. Individuals suffering from KOA commonly experience symptoms such as knee pain, restricted movement, joint stiffness, muscle weakness, impaired mobility, and balance issues [2]. Projections indicate that the number of affected individuals worldwide will increase to 130 million by 2050, placing a substantial burden on healthcare systems globally [3]. Healthcare providers often resort to nonsteroidal anti-inflammatory drugs and intra-articular injections to alleviate pain and delay surgical intervention [4]. However, prolonged pharmacological therapy frequently leads to adverse effects such as peptic ulcers and gastrointestinal bleeding, underscoring the need for alternative, non-pharmacological interventions [4]. Recognizing the limitations of pharmacotherapy, non-pharmacological modalities like physiotherapeutic rehabilitation have gained traction as viable alternatives in KOA management, as endorsed by international guidelines and supported by empirical evidence [5–8]. However, challenges persist in achieving optimal outcomes, with factors such as patient adherence to exercise regimens and the persistence of pain acting as barriers to effective treatment [9, 10].

In this context, acupuncture, with a history spanning over three millennia and originating from China, has garnered attention for its potential in managing chronic musculoskeletal pain [11, 12]. Recent studies have contributed to the growing body of evidence supporting acupuncture’s efficacy in alleviating pain, especially in musculoskeletal disorders [13–15]. Theoretically, acupuncture is believed to elicit the release of endorphins, modulate the nervous system, and induce relaxation, thereby reducing pain signals [16]. Crucially, the effectiveness of acupuncture hinges on the precise selection of acupoints tailored to the individual patient’s physiological condition, highlighting the importance of personalized treatment approaches [16, 17]. While acupuncture holds promise as a safe adjunctive therapy for KOA management, its integration with physiotherapeutic exercise warrants further investigation [11, 14]. The lack of high-quality clinical evidence and standardization in acupuncture techniques and acupoint selection hinder its widespread adoption [6, 11, 14]. Conventionally, acupoint selection has been based on manual methods guided by practitioner expertise, which is highly subjective and reliant on the acupuncturist’s experience and preference [18, 19]. This approach lacks standardization and may introduce variability in treatment outcomes, posing challenges to reproducibility and comparability across studies [18–20].

Moreover, the absence of clear guidelines on acupuncture modalities and acupoint selection further complicates its application in clinical settings [17, 20]. Clinical trials utilizing fixed acupoint protocols have been criticized for disregarding the individualized treatment approaches inherent in traditional Chinese medicine practice [21]. Additionally, only 40% of guidelines issued by conventional medical organizations detail the types of acupuncture, such as manual, electro-acupuncture, or acupressure, and only 5% of these guidelines specify the range of acupuncture points selected [17]. To address this limitation, modern acupuncture has embraced technological innovations such as Acugraph analysis. Acugraph is a device that measures electrical impedance at various points on the body, creating graphical representations that help acupuncturists visualize and analyze the balance and vitality of meridians. This objective assessment of acupoints guides treatment selection [20, 22]. By incorporating technology into acupuncture practice, Acugraph offers a standardized and reproducible approach to acupoint selection, enhancing treatment reliability and consistency [22, 23]. However, more investigation is needed to substantiate the effectiveness of computerized methods of identifying acupoints, particularly in the management of KOA.

In light of these considerations, this study seeks to address the gaps in the existing literature by comparing the effects of computerized point-specific acupuncture, determined by Acugraph, with manual preference acupuncture as adjuncts to physiotherapeutic exercise in KOA management. By rigorously evaluating the synergistic effects of acupuncture and physiotherapy, this research aims to provide valuable insights into optimizing non-pharmacological interventions for KOA.

## Materials and methods

### Study design

**Fig 1:**
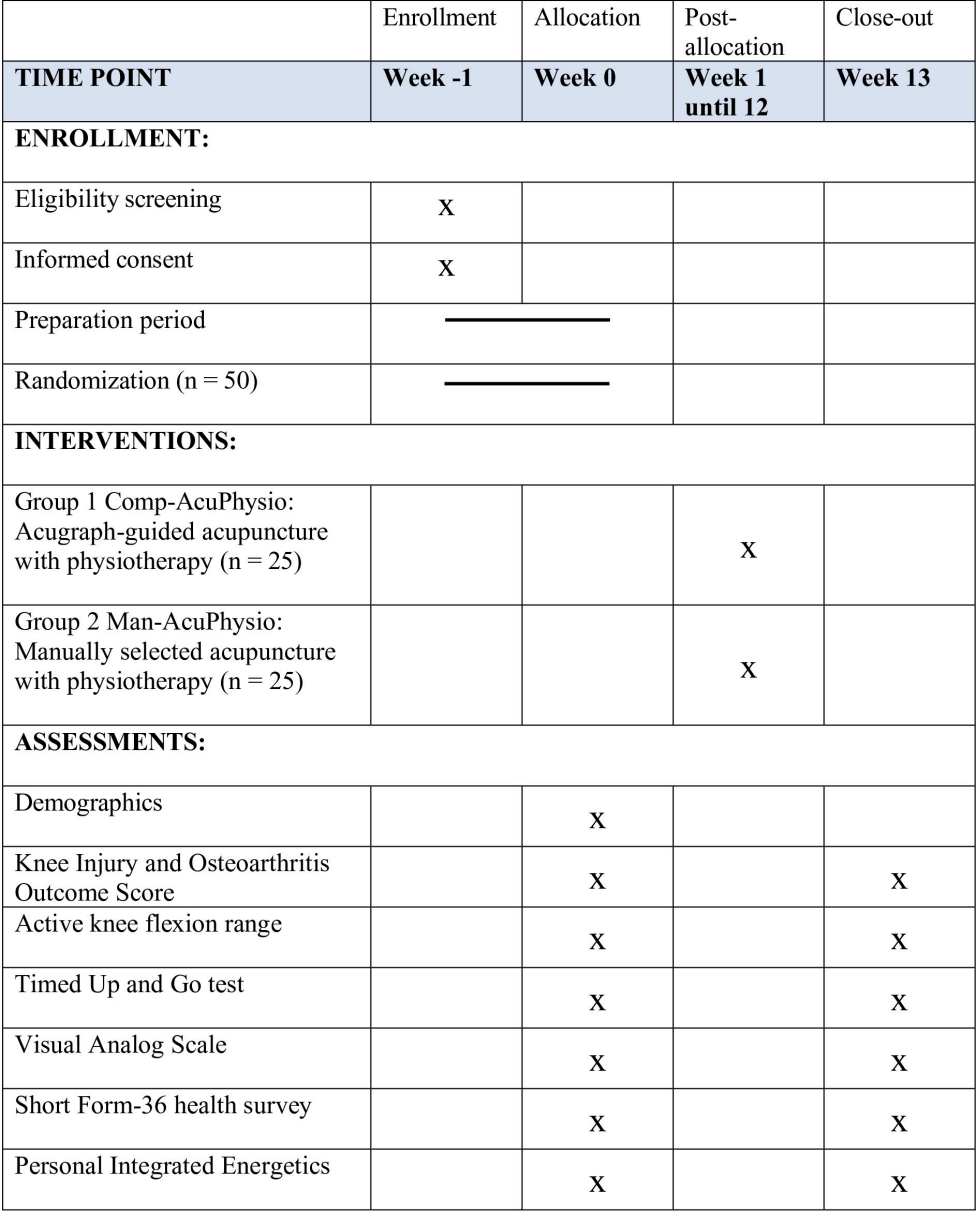
Schedule of enrolment, interventions, and assessments.

This study will utilize a parallel-group (two arms), double-blinded, randomized controlled trial design. The study protocol follows the CONSORT guidelines for clinical trials and meets the recommended criteria outlined in the SPIRIT checklist (S1 File. SPIRIT Checklist). Fig 1 illustrates the details of the schedule for enrollment, interventions, and assessments at each time point.

### Ethical approval and trial registration

Ethical approval was obtained from the Secretariat of the Research Ethics Committee at Universiti Kebangsaan Malaysia (ID: UKM PPI/111/8/JEP-2024-244), approved on 14^th^ June 2024, ensuring compliance with the ethical principles outlined in the Helsinki Declaration (S2 File. Ethical approval letter). The trial protocol has been registered with the Australian New Zealand Clinical Trials Registry (ANZCTR), which is approved under the World Health Organization International Clinical Trials Registry Platform (WHO ICTRP), and the registration number is ACTRN12624000646549p. Any relevant study protocol modifications, along with their justification, will be communicated to and updated with the ethics committee and trial registry.

### Study setting

The study will be conducted at Grand Care Rehab Seremban (Physiotherapy & Acupuncture), Malaysia. Grand Care Rehab Seremban is an established clinical setup that offers conventional physiotherapy rehabilitation services and specializes in complementary medicine approaches, particularly traditional Chinese medicine acupuncture. Recruitment strategies include: (i) utilizing social media and online forums, (ii) collaborating with local organizations and community groups, (iii) implementing referral programs, and (iv) distributing brochures and posters in targeted areas.

### Target population

The inclusion criteria are as follows: (i) adults aged 18 years and above of both genders, (ii) diagnosed with unilateral or bilateral KOA by an orthopedic physician, with radiologic confirmation of KOA grade 2 (mild) or 3 (moderate) according to the Kellgren-Lawrence classification based on an X-ray performed within the last 12 months, and (iii) capable of participating in the intervention and assessment program without restrictions. In cases of bilateral KOA involvement, the more painful knee will be selected for outcome evaluation. The exclusion criteria include: (i) those with recent knee trauma, ligament damage, fracture, or surgery within the past 6 months, (ii) a history of local knee tumor or malignancy, recent prolotherapy, hyaluronic acid, or corticosteroid injections within the past 6 months, and (iii) individuals who oversensitive to needles.

### Sample size calculation

The sample size was calculated using G*Power software version 3.1.9.7, with a focus on achieving a robust estimate for detecting clinically meaningful changes. Specifically, a minimum clinically important difference (MCID) of 11.1 points, with a standard deviation (SD) of 3.8 points, was used based on established values for the primary outcome, the total Knee Injury and Osteoarthritis Outcome Score (KOOS) [24]. This estimation considered a power of 80%, an effect size of 0.2, a significance level of 5%, and an anticipated dropout rate of 20% [25, 26]. Consequently, the total calculated sample size was 50 participants, evenly divided into two groups of 25 participants each. KOOS was selected as the primary outcome measure for this study due to its comprehensive assessment of key domains relevant to KOA, including pain, symptoms, activities of daily living, sport and recreation, and quality of life [27]. The tool has been widely validated in populations with KOA and is sensitive to changes following interventions, making it an appropriate and robust measure for capturing patient-reported outcomes in this context [27].

### Screening, recruitment, and randomization

**Fig 2:**
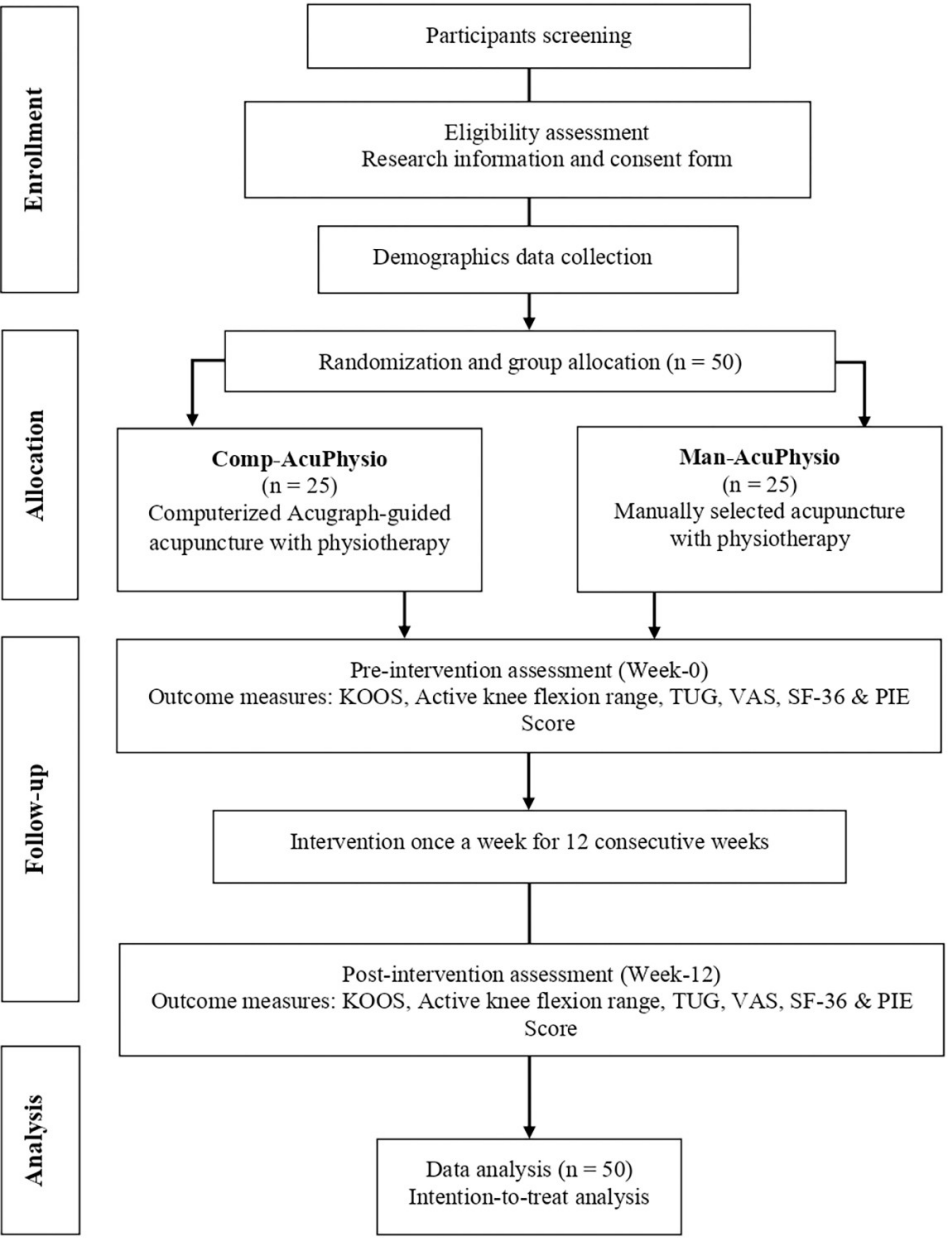
Study flowchart.

Eligible participants will be screened by a research assistant who is not involved in the intervention or outcome assessment. Those who meet the criteria will be provided with verbal and written information, followed by obtaining signed informed consent. Sociodemographic details will be collected, and each participant will be assigned a unique identification number to safeguard privacy and confidentiality. Participants (n = 50) will be randomly allocated to one of two groups at a 1:1 ratio: (i) Group 1 Comp-AcuPhysio (n = 25) which uses a computerized method of acupoint selection as an adjunct to physiotherapy, or (ii) Group 2 Man-AcuPhysio (n = 25) which uses a manual method of acupoint selection as an adjunct to physiotherapy. The randomization will be performed using a computerized simple block randomization method. The randomization process will be conducted by the principal investigator, who is not involved in the intervention or outcome assessment. Allocation concealment will be maintained through sealed opaque envelopes describing the treatment group. Fig 2 illustrates the study flowchart.

### Group allocation and intervention

Participants in both groups will undergo standardized physiotherapy rehabilitation, delivered through closely supervised sessions. The protocols for these exercises will be adapted from previous relevant studies, ensuring alignment with established practices [9, 28–30]. Exercise prescriptions and progressions will be modified according to the individual needs and impairments of each participant. Examples of the exercise components are illustrated in Table 1. In addition, strategies to improve and monitor adherence to intervention protocols, such as personalized feedback and regular follow-ups, will be implemented. Monitoring procedures will include attendance tracking, session logs, and periodic assessments to ensure participants are following the prescribed regimen.

**Table 1:** Rehabilitation exercises.

Exercise	Prescription
Range of motion	Prone knee bend; 2 sets, 10 repetitions each Supine alternate knee bend; 2 sets, 10 repetitions
Stretching	Standing quadriceps stretch; 1 set, 3–5 repetitions, 15-second hold each Calf stretch in long sitting; 1 set, 3–5 repetitions, 15-second hold each Supine hamstring stretches; 1 set, 3–5 repetitions, 15-second hold each
Strengthening	Sitting knee extension; Week 1–2, 1 set, 5 repetitions, 5-second hold; Week 3–5, 1 set, 7–10 repetitions, 5-10-second hold; Week 6–8, 2 sets, 10 repetitions, 10-second hold Supine straight leg raises; Week 1–2, 1 set, 5 repetitions, 5-second hold; Week 3–5, 1 set, 7–10 repetitions, 5-10-second hold; Week 6–8, 2 sets, 10 repetitions, 10-second hold Static quadriceps; Week 1–2, 1 set, 5 repetitions, 5-second hold; Week 3–5, 1 set, 7–10 repetitions, 5-10-second hold; Week 6–8, 2 sets, 10 repetitions, 10-second hold Side-lying straight leg raise; Week 1–2, 1 set, 5 repetitions, 5-second hold; Week 3–5, 1 set, 7–10 repetitions, 5-10-second hold; Week 6–8, 2 sets, 10 repetitions, 10-second hold

In addition to the standard physiotherapy rehabilitation, participants in this study will receive acupuncture treatment according to their assigned group: Group 1 Comp-AcuPhysio (n = 25) or Group 2 Man-AcuPhysio (n = 25). The acupuncture treatment protocols are adapted from previous relevant studies [13, 14, 31, 32]. Both acupuncture and physiotherapy treatments for both groups will be administered by a single qualified physiotherapist who is also an acupuncturist and is not involved in outcome assessment. A standard acupuncture session for the treatment of KOA will involve the use of approximately 4 to 12 acupuncture points [13, 32]. Needles will be inserted to appropriate depths (25mm or 40mm), with penetration ranging from 0.8 to 3 cm, and left in place for 20 minutes [13, 32]. The details of the respective group interventions are as follows:

#### Group 1 Comp-AcuPhysio

**Fig 3:**
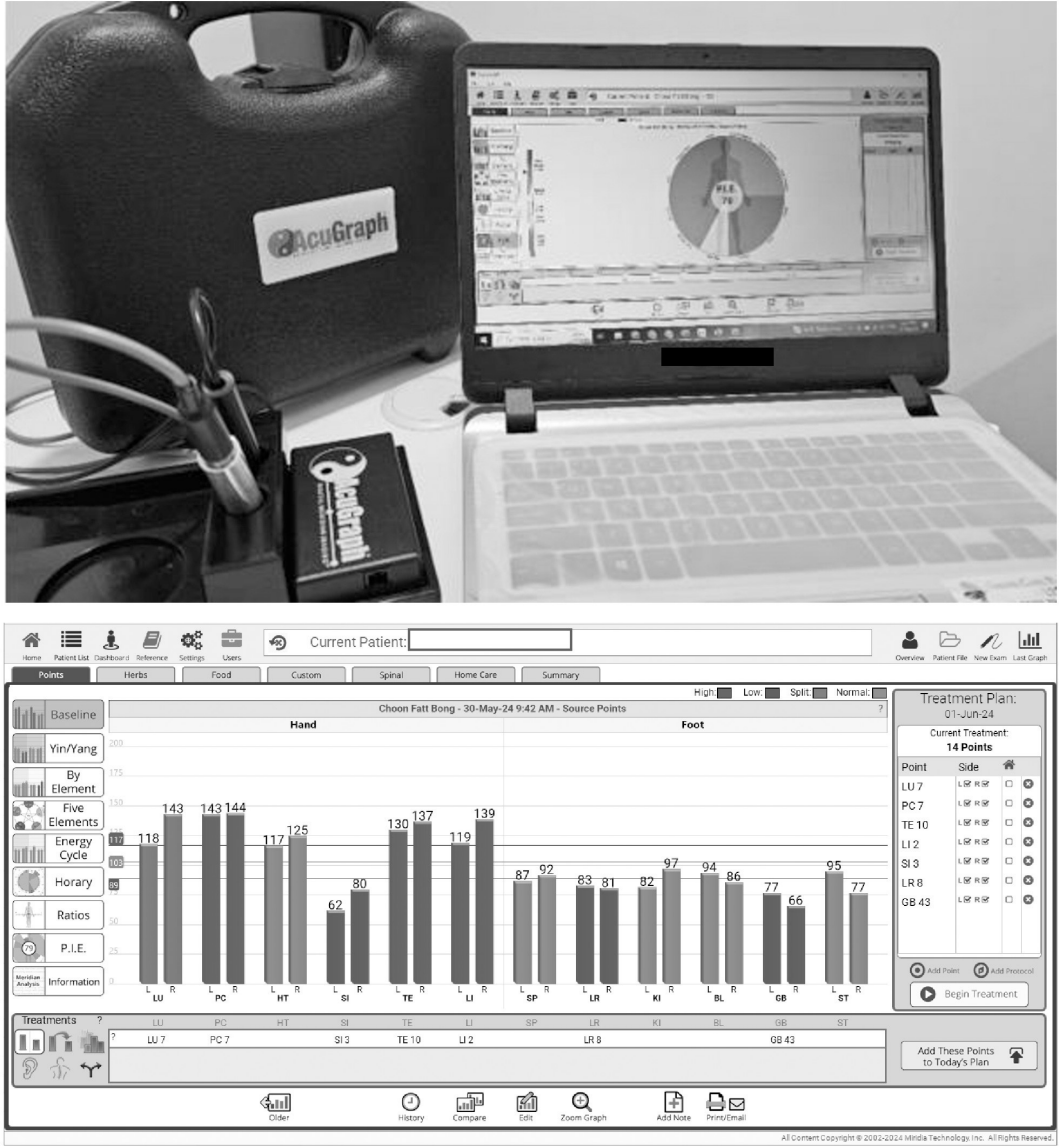
Computerized acupuncture point identification using Acugraph system.

Participants in this group will receive a 20-minute acupuncture treatment based on a computerized method for identifying point-specific acupuncture using the AcuGraph system (Fig 3), combined with 40 minutes of personalized physiotherapy, totaling 60 minutes per session. The sequence of the interventions will be randomized, and the interventions will be administered once a week for 12 consecutive weeks. Initially, participants will be screened using the AcuGraph system, an electrodermal screening device that assesses energy flow and stress levels by measuring electrical conductance at specific acupuncture points on the skin [33]. This analysis provides information into overall health and meridian imbalances, which are crucial for locating precise acupuncture points on the body [33]. This ensures accurate and effective needle placement, ultimately enhancing the precision of acupuncture treatments to optimize therapeutic outcomes by targeting the body’s vital energy pathways. The computer-generated acupoints specific to each participant will guide the precise points of treatment [33].

#### Group 2 Man-AcuPhysio

Participants in this group will receive a combined treatment of acupuncture and personalized physiotherapy, lasting a total of 60 minutes per session. Each acupuncture treatment will be approximately 20 minutes long, tailored according to the acupuncturist’s manual preferences. To ensure blinding of participants, a placebo Acugraph analysis will be used to conceal group allocation. Based on acupuncturist’s manual preferences, commonly used acupoints include Yanglingquan (GB34), Yinlingquan (SP9), Dubi (ST35), and Neixiyan (EX-LE4) [34]. Depending on the patient’s condition, the acupuncturist may add two or more local acupuncture points based on their professional judgment (Table 2) [34]. The acupuncturist will conduct a thorough assessment of each patient’s condition, considering any contraindications and maintaining a sterile environment. The acupuncture points selected will specifically target knee pain and arthritis [13, 32]. These points may vary based on the pathway of Qi flow and include both proximal and distal points around the antero-medial and antero-lateral parts of the knee joint [13, 32]. Additionally, acupoints located on the stomach and spleen meridian channels, which pass through the knee area, may be used [13, 32].

**Table 2:** Examples of manual acupuncture points selection.

Acupoint	Name	Location
Fixed acupoints	Yanglingquan (GB34)	On the lateral side of the lower leg, in the depression anterior and inferior to the head of the fibula
	Yinlingquan (SP9)	On the medial side of the shank, at the depression posterior and inferior to the medial condyle of the tibia.
	Dubi (ST35)	Between the lower edge of the patella and the upper tip of the tibia, at the midpoint of the patella ligament
	Neixiyan (EX-LE4)	In the depression located on the medial side of the patellar ligament
Optional: anteromedial parts knee joint	Xuehai (SP10)	On the anterolateral aspect of thigh, on the bulge of vastus medialis muscle, 2 cun superior to the medial end of the base of patella
Optional: anterolateral parts knee joint	Liangqiu (ST34)	On the anterolateral aspect of thigh, between vastus lateralis muscle and lateral border of rectus femoris tendon, 2 cun superior of the base of patella
Optional: stomach meridian channels that pass through the knee area	Zusanli (ST36)	3 cun directly below ST35, and one finger breadth lateral to the anterior border of tibial
	Fenglong (ST40)	On the anterolateral aspect of leg, lateral border of tibialis anterior muscle, 8 cun superior to the prominence of lateral malleolus
Optional: spleen meridian channels that pass through the knee area	Sanyinjiao (SP6)	On the tibial aspect of the leg, posterior to the medial border of the tibial, 3 cun superior to the prominence of medial malleolus
	Gongsun (SP4)	On the medial aspect of the foot, anteroinferior to the base of the 1^st^ metatarsal bone, at the border between red and white flesh

Note: 1 cun (~20 mm) is the width of the interphalangeal joint of the patient’s thumb.

### Blinding

Screening, recruitment, and randomization of participants will be conducted by a research assistant uninvolved in the intervention or outcome assessment, ensuring unbiased processes [35]. Outcomes will be assessed by a blinded assessor not involved in the intervention. The James Blinding Index (JBI) will be used to evaluate blinding success from the participants’ perspective [35].

### Safety consideration

Adverse events related to acupuncture, including pain, bleeding, fainting, or other severe incidents, will be promptly addressed and meticulously documented in the case report form [14]. Strict safety protocols will be adhered to throughout the procedure, including: (i) ensuring treatments are administered by qualified acupuncturists and therapists to guarantee safe and proficient delivery, and (ii) implementing rigorous infection control measures. These measures encompass hand hygiene, the use of disposable needles, proper skin preparation, maintenance of a sterile environment, appropriate needle handling, glove usage, and thorough patient screening [36, 37]. In addition, interim analyses will be conducted at predefined intervals to monitor safety and efficacy. These analyses will evaluate the incidence of adverse events and the overall effectiveness of the treatment. Any relevant incident will be discussed among the research team, and decisions will be made based on consensus. Recommendations regarding the continuation, modification, or termination of the trial will be based on predefined criteria, including unacceptable adverse event rates or clear evidence of benefit or harm.

The safety of acupuncture in the management of musculoskeletal pain has been well-documented in the literature, with minimal adverse events reported [14, 15, 38]. Therefore, a data and safety monitoring committee (DSMC) will not be appointed for this trial, given its low-risk nature. This approach is consistent with comparable trials such as those conducted by Wang et al. (2024) and Pang et al. (2022), where no DSMC was deemed necessary in randomized studies of acupuncture for KOA [39, 40]. Nonetheless, all safety events, including adverse effects, will be carefully recorded, and participant monitoring will remain thorough to ensure ongoing safety.

### Outcome measures and assessment interval

Outcome measures will be assessed at two intervals: (i) baseline (pre-intervention) and (ii) immediately post-intervention. All data, including consent forms, sociodemographic information, and outcome measures, will be collected using hardcopy clinical research forms (CRFs). Data from the CRFs will be transferred into an Excel spreadsheet for storage and further analysis. All hardcopy documents will be kept in a secure location, and electronic files will be protected on a password-secured computer with restricted access. These research records, in both physical and electronic formats, will be retained for a minimum of five years following the study’s publication.

**Fig 4:**
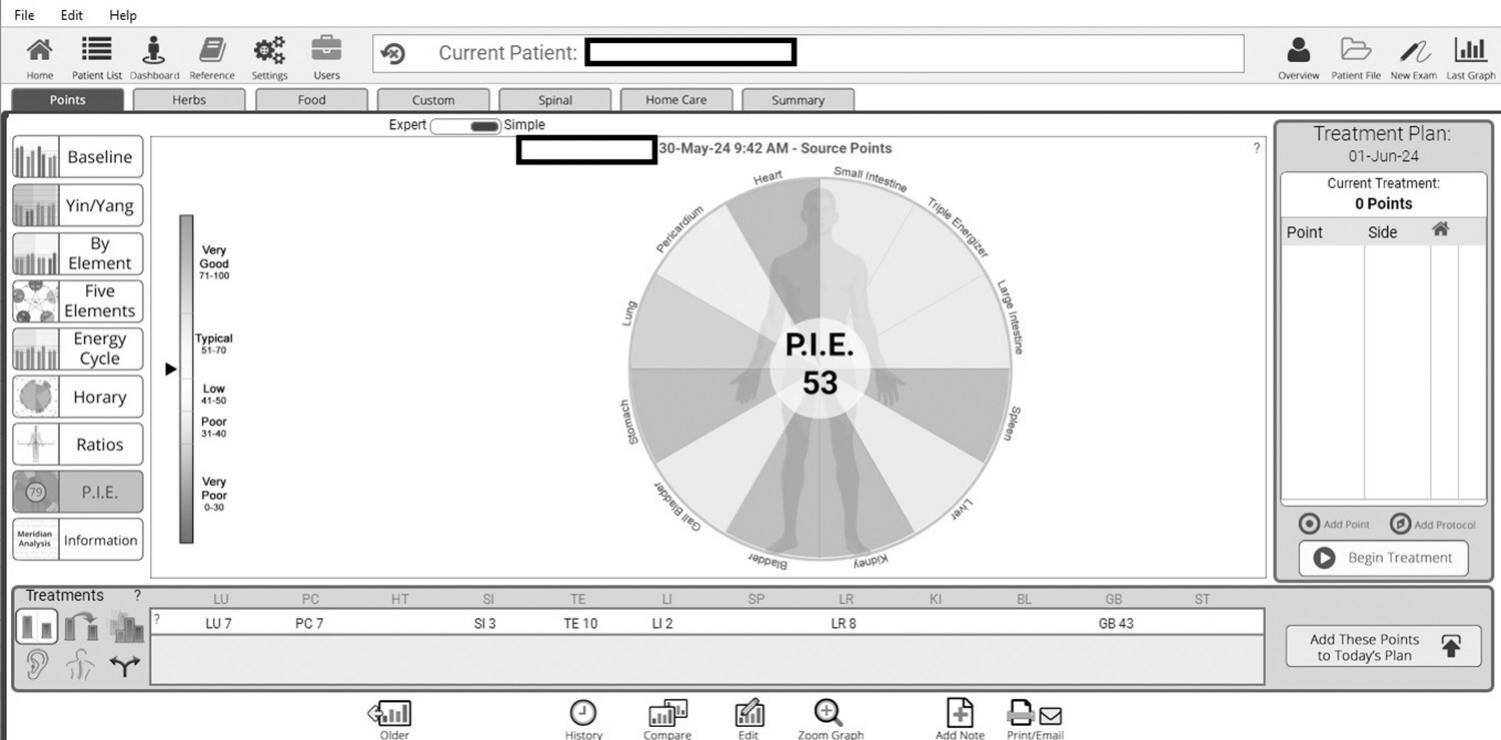
Example of a Personal Integrated Energetics score based on an analysis using the Acugraph system.

- **Knee Injury and Osteoarthritis Outcome Score (KOOS):** The KOOS questionnaire will be administered to evaluate participants’ perceptions of knee function and osteoarthritis-related symptoms. It consists of five subscales: pain (nine items), symptoms (seven items), activities of daily living function (17 items), sport and recreation function (five items), and quality of life (four items) [27]. The test-retest reliability of KOOS is excellent, with intraclass correlation coefficients ranging from 0.91 to 0.99 [27]. Each item is scored on a five-point Likert scale ranging from 0 (no problems) to 4 (extreme problems). The individual subscale scores are calculated by summing the responses and transforming the total into a 0–100 scale. A score of 0 indicates severe knee-related issues, while a score of 100 reflects no knee-related problems [24, 27]. Apart from calculating the total KOOS score, individual subscales for each of the domains will be calculated and analyzed separately to provide a detailed assessment of different aspects of knee function [24]. This approach allows us to capture domain-specific improvements or changes in response to the intervention, ensuring a comprehensive evaluation of KOA outcomes [24].
- **Active knee flexion range of motion (AKF):** Knee flexion will be measured using a long-arm universal goniometer. The stationary arm of the goniometer will be aligned with the greater trochanter along the outer thigh, and the other arm with the lateral malleolus of the ankle. The goniometer has a high reliability value, with an intraclass correlation coefficient of 0.997 for knee flexion in subjects with knee impairments [41]. Normal knee flexion ranges from 0 degrees (knee in full extension) to approximately 140 degrees (maximal knee flexion). Higher ROM values within this range reflect greater knee flexion capacity, whereas lower values suggest restricted knee movement [42].
- **Timed Up and Go Test (TUG):** The TUG test assesses functional mobility by measuring the time taken to stand up from a chair, walk 3 meters, turn, and return to sit down [43]. In this study, the average time of three trials will be documented to improve accuracy. The time taken to complete the task is recorded, with shorter times indicating better functional mobility, balance, and lower fall risk [43]. A completion time exceeding 14 seconds suggests significant impairment in functional mobility and is associated with an elevated risk of falls [43]. The minimal detectable change for this test is 1.10 seconds [43]. The TUG test demonstrates high reliability and validity for mild to moderate KOA, with intra-rater and inter-rater reliability values of 0.97 (95% CI, 0.95–0.98) and 0.96 (95% CI, 0.94–0.97), respectively [43].
- **Visual Analog Scale (VAS):** The VAS is a subjective measurement tool commonly used to assess pain intensity. It is determined using a linear scale spanning from 0 to 10 centimeters. On this scale, 0 indicates the absence of pain, while 10 signifies severe pain, with varying degrees of pain intensity in between of it [44]. Participants indicate their pain levels by marking positions along the line. The reliability of the VAS for assessing chronic pain in clinical settings is excellent, with a test-retest intraclass correlation coefficient of 0.97 [44].
- **The Short Form 36 Health Survey (SF-36):** The Short Form 36 Health Survey (SF-36): The SF-36 Health Survey (SF-36) is a comprehensive questionnaire consisting of 36 items designed to assess various aspects of health and well-being, covering eight primary domains of overall health status. Patients are asked to respond based on their recent experiences, typically over the past week, providing a snapshot of their current health [45]. Response options include formats such as “yes/no,” “never/sometimes/often/always,” or Likert scales ranging from “excellent” to “poor.” Each domain is scored from 0 to 100, with higher scores indicating better health or functioning in that area [45]. The domains are as follows: (i) physical functioning, (ii) role limitations due to physical health, (iii) role limitations due to emotional problems, (iv) energy levels and fatigue, (v) psychological well-being, (vi) social functioning, (vii) pain intensity and interference with activities, and (viii) overall health perceptions [45]. The SF-36 also provides two overarching summary scores: the Physical Component Summary (PCS) and the Mental Component Summary (MCS), which represent the physical and mental health dimensions, respectively [45]. Higher scores in these summaries denote better overall health status in the respective areas.
- **Personal Integrated Energetics—Acugraph System:** This system measures and analyzes the energetic status of acupuncture meridians. Each wedge of the circle in the Personal Integrated Energetics (PIE) chart represents one meridian pathway, where those shown in green are balanced and functioning properly [33]. The PIE score includes a complete analysis of all meridian pathways and reflects the overall health of the meridian energy system (Fig 4) [33]. A perfect score of 100 reflects complete balance and unimpeded flow of Qi energy, while a lower score indicates various imbalances that need to be addressed [33].

### Data analysis

Data entry and storage will follow strict protocols to ensure quality and confidentiality. Double data entry will be employed to reduce errors, and range checks will be conducted to detect any outliers or inconsistencies [46]. All data will be encrypted and stored in a password-protected system with limited access, complying with privacy regulations [46]. The presentation and reporting of the study’s findings will adhere to CONSORT reporting guidelines, ensuring transparency and consistency in the reporting of the trial. The data analysis will be conducted by a researcher who is blinded to group allocation, and in consultation with an expert statistician. Measurement data will be tested for normality. If data conform to a normal distribution, they will undergo t-test analysis, while non-normally distributed data will be analyzed using the Mann-Whitney U test. For normally distributed data, the mean and standard deviation will be calculated, and for non-normal data, the median and percentages will be presented.

In this study, an intention-to-treat analysis will be employed. This approach will include all eligible randomised participants initially allocated to the study, regardless of their adherence to the protocol. ITT analysis is chosen because it preserves the benefits of randomization, maintains the baseline comparability of groups, and provides a more conservative estimate of the treatment effect, thereby increasing the external validity of the findings [47]. Missing data will be handled using the multiple imputation technique, which involves creating multiple datasets by imputing missing values through statistical models [48]. The results from these datasets will then be combined to account for the uncertainty caused by the missing data [48].

Additionally, a per-protocol analysis will be conducted to compare findings from participants who adhered strictly to the protocol [47]. This comparison will help assess any differences in outcomes between the two approaches [47]. To examine differences between the both groups over time, a repeated measures ANOVA will be employed to assess changes in KOOS, AKF, TUG, VAS, SF-36, and PIE scores over time (time main effect) and to evaluate whether these changes over time differed between the two groups (group-by-time interaction). Additionally, to assess the extent of variation between groups, the effect size of each variable will be calculated using Cohen’s d, where values of 0.2, 0.5, and 0.8 indicate small, medium, and large effects, respectively [26]. In addition, subgroup analysis will be performed based on key factors such as age groups, BMI, and disease severity (mild vs. moderate), as these variables are considered important contributors to outcomes in KOA. The analysis will help identify whether the intervention effects vary across these subgroups.

## Results

The screening and recruitment of participants are expected to commence by July 2024. Data collection is projected to conclude by July 2025. The trial findings will be disseminated through publications and data sharing agreements.

## Discussion

Despite accumulating evidence supporting acupuncture’s efficacy, its integration with physiotherapy for KOA remains constrained by the lack of robust evidence. Key challenges include the absence of standardized acupuncture protocols, marked by considerable variations in techniques and acupoint selection, which complicates the interpretation of existing studies [19, 20]. Moreover, a systematic review focusing on musculoskeletal pain noted the absence of consensus regarding the rationale behind selecting specific acupoints, whether local or distant [49]. These issues underscore the necessity for well-designed clinical trials that incorporate Acugraph, a computerized acupoint selection tool, in conjunction with physiotherapy for KOA. Such trials aim to establish standardized protocols and generate high-quality evidence for clinical application. This study seeks to compare the efficacy of computerized versus manual methods in identifying acupoints specific to acupuncture as an adjunct to physiotherapy for managing KOA.

Anticipated outcomes include demonstrating the effectiveness of both interventions in reducing pain and enhancing functional outcomes, with the computerized Acugraph-guided method potentially showing more favorable results. This expectation is consistent with previous findings suggesting that integrating technology into acupuncture practice enhances treatment precision and overall outcomes [22]. The objective and reproducible nature of Acugraph may mitigate variability inherent in manual acupuncture, thereby standardizing treatment protocols and enhancing reliability across diverse practitioners and settings [23]. Furthermore, the study aims to underscore the significance of personalized treatment in KOA management. The individualized approach facilitated by Acugraph-guided acupuncture, which tailors acupoint selection based on the patient’s unique meridian imbalances, is anticipated to contribute to improvements in pain relief and knee function. This personalized approach may prove more effective compared to conventional manual acupuncture, which often relies on the subjective judgment and experience of the practitioner [34]. These expected findings emphasize the relevance of precision medicine in managing chronic conditions, particularly in KOA where patient-specific factors significantly influence treatment outcomes [50].

Another pivotal aspect of anticipated findings involves the integration of acupuncture with physiotherapy. Previous research has suggested that acupuncture complements physiotherapy by alleviating pain and facilitating better engagement in physical exercises [51]. This synergistic effect may arise from acupuncture’s modulation of pain pathways and reduction of inflammation, thereby complementing the mechanical benefits of physiotherapy [16]. Lastly, the expected outcomes of this study carry substantial implications for clinical practice and future research. The adoption of computerized methods such as Acugraph in acupuncture not only offers a standardized approach but also enhances treatment objectivity and reproducibility, thereby enhancing its utility in clinical settings. It is anticipated that this study’s findings will contribute to the expanding body of evidence supporting the integration of technology into traditional medical practices, paving the way for more effective and personalized treatment strategies in managing KOA.

## Conclusion

This study underscores the value of integrating Acugraph-guided acupuncture with physiotherapy to enhance precision and outcomes in KOA management. By standardizing acupoint identification, Acugraph addresses variability in manual methods, supporting a more personalized approach. The anticipated findings could drive advancements in precision medicine, optimizing non-pharmacological interventions for KOA and enhancing patients’ pain relief and functional outcomes.

## Supporting information

S1 File SPIRIT checklist. (PDF)

S2 File Ethical approval letter. (PDF)
