# Supplementary material for: Comparing the effects of computerized versus manual methods of identifying point-specific acupuncture as an adjunct to physiotherapy in the management of knee osteoarthritis: A randomized controlled trial protocol
Source: PLoS One. 2025 Jan 13;20(1):e0313761. doi: 10.1371/journal.pone.0313761 (PMC11730376; doi:10.1371/journal.pone.0313761)
Supplement: S2 File — (PDF) [file pone.0313761.s002.pdf]

Reference : UKM PPI/111/8/JEP-2024-244
Date : 14 June 2024

Dr. Mohd Azzuan Bin Ahmad
Center for Rehabilitation and Special Needs Studies (ICaRehab)
Faculty of Health Sciences
The National University of Malaysia

Dear Professor/Datuk/Dato'/Datin/Sir/Madam,

ETHICAL APPROVAL TO CONDUCT RESEARCH IN THE NATIONAL UNIVERSITY OF MALAYSIA

Research title	:	Psychophysical Effects Of Computerised Point-Specific Acupuncture As Adjunctive To Physiotherapy In Knee Osteoarthritis: Mixed Methods Research Design
Ethics Ref. No.	:	JEP-2024-244
Approval period	:	12 June 2024 – 11 June 2026
Study Site	:	Grand Care Rehab Seremban, Negeri Sembilan, Malaysia
Sample Size	:	50 participants (25 participants for each group)

With reference to the above.

2. The Research Ethics Committee, The National University of Malaysia (RECUKM) has provided ethical approval for above study. Please be reminded permission from the Deputy Dean of research of the faculty or Director of Institute/Centre and all relevant heads of departments / units where the study will be carried out must be obtained prior to the study. You are required to follow and comply with their decision and all other relevant regulations.

3. Please note that the investigator's responsibility is to ensure that:

i. All Adverse Events should be reported to RECUKM as soon as possible.
ii. Progress report should be submitted every **6 Months.**
iii. All changes / amendments to the study documents / study sites / study team must be notified to the RECUKM. All changes must be approved by RECUKM before continuation of the study.
iv. Application for renewal of the approval has to be submitted to RECUKM within one month (1 month) prior to the expiry of ethical approval.
v. Final Report should be provided to RECUKM when the project is complete.
vi. Please take note that all records and data are to be kept strictly CONFIDENTIAL and can only be used for the purpose of this study. All precautions are to be taken to maintain data confidentiality.

Required forms can be obtained from the The Research Ethics Committee, The National University of Malaysia (RECUKM) website: **https://www.ukm.my/jepukm**

Thank you.

Yours sincerely,

Chairman
Research Ethics Committee
The National University of Malaysia

c.c.

- **Deputy Dean (Research & Innovation)**
  Faculty of Health Sciences
  National University Of Malaysia

- **Chairperson of Center for Rehabilitation and Special Needs Studies (ICaRehab)**
  Faculty of Health Sciences
  National University Of Malaysia

- **Dato Dr. Jeals Tan Chee Hou**
  CEO of Grand Care Rehabilitation

- **Lee Chai Li (P137866)**
  Center for Rehabilitation and Special Needs Studies (ICaRehab)
  Faculty of Health Sciences
  National University of Malaysia

- Approval letter File 2024

SEKRETARIAT ETIKA PENYELIDIKAN UKM • *UKM RESEARCH ETHICS COMMITTEE*

**NAME OF ETHICS COMMITTEE/IRB:**<br>Research Ethics Committee,<br>The National University of Malaysia	**ETHICS COMMITTEE/IRB REF NO :**<br>**UKM PPI/111/8/JEP-2024-244**

PROTOCOL TITLE:

Psychophysical Effects Of Computerised Point-Specific Acupuncture As Adjunctive To Physiotherapy In Knee Osteoarthritis: Mixed Methods Research Design

PRINCIPAL INVESTIGATOR:

Dr. Mohd Azzuan Bin Ahmad
Center for Rehabilitation and Special Needs Studies (ICaRehab)
Faculty of Health Sciences
The National University of Malaysia

The following items [X] have been received and reviewed in connection with the above study to be conducted by the above investigator.

Documents

- [X] Research Application Form (UKM-JEP-BO01)
- [X] Review Form (UKM-JEP-BO02)
- [X] Non-Disclosure Agreement (UKM-JEP-BO03)
- [X] Conflict Of Interest Form
- [X] Research Proposal / Protocol
- [X] Participant Information Sheet:
  - [X] Malay [X] English [ ] Others :
- [X] Participant Consent Form:
  - [X] Malay [X] English [ ] Others :
- [X] Questionnaire:
  - [ ] Malay [X] English [ ] Others :
- [ ] Case report Form:
  - [ ] Malay [ ] English [ ] Others :
- [X] Curriculum Vitae of Researchers:
  - [X] Principal [X] Co-researcher [X] Student
- [X] Good Clinical Practice Certificate (GCP)
- [ ] Project Agreement

The Research Ethics Committee, The National University of Malaysia operates in accordance to the International Conference of Harmonization Good Clinical Practice Guidelines.

Comments (if any): --

Date of Approval: 12 June 2024

Sekretariat Etika Penyelidikan Universiti Kebangsaan Malaysia
Tingkat 1, Blok Klinikal, Hospital Canselor Tuanku Muhriz,Pusat Perubatan UKM, Jalan Yaacob Latif, Bandar Tun Razak, 56000 Cheras Kuala Lumpur.
Telefon: +603-9145 5046 / 5048
Email: sepukm@ukm.edu.my Web: https://www.ukm.my/jepukm/

RESEARCH PROPOSAL

TITLE:

PSYCHOPHYSICAL EFFECTS OF COMPUTERISED POINT-SPECIFIC ACUPUNCTURE AS ADJUNCTIVE TO PHYSIOTHERAPY IN KNEE OSTEOARTHRITIS: MIXED METHODS RESEARCH DESIGN

RESEARCHER:

DR MOHD AZZUAN AHMAD (PRINCIPAL INVESTIGATOR)

PHYSIOTHERAPY PROGRAM, CENTRE FOR REHABILITATION AND SPECIAL NEEDS STUDIES, FACULTY OF HEALTH SCIENCES, UKM

Executive Summary

Knee osteoarthritis (KOA) stands as a significant contributor to disability, underscoring the need for more effective rehabilitation methods to enhance clinical outcomes. Due to its chronic degenerative nature, healthcare professionals frequently opt for a pharmaceutical strategy. However, extended pharmacological treatment often leads to side effects, such as peptic ulcers and gastrointestinal bleeding. Coventional therapy fall short, thus the exploration of alternative treatments, such as therapeutic acupuncture, has emerged as a promising avenue. However, uncertainties persist regarding the efficacy of combining acupuncture with physiotherapy, prompting a call for more comprehensive research to address existing gaps in knowledge. This study aims to fill these gaps by investigating both short-term (immediately post-intervention) and long-term (3 months after the last intervention) effects of a computerized method for identifying point-specific acupuncture generated by the Acugraph system when combined with physiotherapy on knee pain, physical function, psychological well-being, and health perception. Additionally, the research seeks to examine the correlation between improvements in KOA physical function, assessed through established tools like KOOS and TUG, and the AcuGraph Personal Integrated Energetics (PIE) score which serve as an indicator of the overall health of the meridian energy system. This study will employ a mixed-mode research design which will be conducted in three phases. The first two phases involve a randomized controlled trial with 40 participants. In Group 1 (Comp-AcuPhysio: n = 20), participants will receive acupuncture treatment based on the computerized method generated by the Acugraph system, combined with personalized physiotherapy. In Group 2 (Man-AcuPhysio: n = 20), participants will receive acupuncture treatment based on manual preferences of the acupuncturist, also combined with personalized physiotherapy. Each group will undergo 60-minute sessions once a week for 12 weeks. Repeated measures of ANOVA will be employed for data analysis. The third phase focuses on the qualitative component, utilizing structured one-on-one interviews to explore participants' psychological well-being, experiences, and perspectives. This qualitative aspect aims to provide depth and context to the quantitative findings. The anticipated findings are expected to provide a better understanding of the holistic treatment impact and patient experiences in terms of well-being and the long-term effects of combining computerized point-specific acupuncture with physiotherapy.

Keywords: Knee Osteoarthritis; Exercise; Acupuncture; Computerized acupoints; Psychophysical Responses; Pain; Health Perception

Table of Contents

1.0 Introduction ... 6
1.1 Study Background ... 6
1.2 Problem Statement ... 8
1.3 Research Questions ... 9
1.4 Research Objectives ... 9
1.4.1 General objectives: ... 9
1.4.2 Specific objectives: ... 9
1.5 Research Hypothesis ... 10
1.6 Research Significance ... 10
1.7 Research Framework ... 12
2.0 Literature Review ... 13
2.1 Pathophysiology of KOA ... 13
2.2 Prevalence and Impact of KOA ... 13
2.3 Current Management of KOA ... 15
2.4 Acupuncture as An Alternative Management in Musculoskeletal Disorder ... 16
2.5 Clinical Evidence of Acupuncture Therapy In KOA ... 16
2.5.1 Effects of Acupuncture on Pain ... 17
2.5.2 Effects of Acupuncture on Physical Function ... 17
2.5.3 Effects of Acupuncture on Psychological Well-Being ... 18
2.5.4 Safety and Adverse Effects of Acupuncture ... 18
2.6 AcuGraph in Acupunture Application ... 19
2.7 Summary of Literature Findings ... 20
3.0 Methodology ... 26
3.1 Phase 1: Quantitative Design - Randomized Controlled Trial (Short-Term Outcomes) 26
3.1.1 Study Design ... 26
3.1.2 Study Settings ... 26
3.1.3 Target Population ... 26
3.1.4 Sample Size Calculation ... 27
3.1.5 Sampling and randomization ... 27
3.1.6 Group allocation and intervention ... 28

3.1.7 Outcomes Measures 31
3.1.8 Data Analysis 33
3.2 Phase 2: Quantitative Design - Randomized Controlled Trial (Long-Term Outcomes) 33
3.3 Phase 3: Qualitative Study - Structured One-On-One Interviews (Participants' Psychological Well-being, Experiences, And Perspectives) 34
3.3.1 Procedures 34
3.3.2 Data Analysis 35
3.4 Gantt Chart 38
4.0 References 39

1.0 Introduction

1.1 Study Background

Knee osteoarthritis (KOA) represents a formidable global challenge, imposing significant disability and an urgent need for effective rehabilitation (Alghadir & Khan, 2022; Kamsan et al., 2020). Conventional methods often fall short, prompting exploration into alternative therapies like therapeutic acupuncture (Rongrong Li et al., 2020; Tian et al., 2022). However, uncertainties persist regarding the efficacy of integrating acupuncture with physiotherapy, necessitating a thorough investigation. This study examine (i) the effects of computerized point-specific acupuncture that determined by Acugraph system paired with physiotherapy on knee pain, physical function, psychological well-being, and health perception in the short term and long term, and (ii) the correlation between improvements in KOA physical function (assessed through well-established tools like KOOS and TUG) and the AcuGraph Personal Integrated Energetics (PIE) score, which serves as an indicator of the overall health of the meridian energy system. The existing literature emphasizes the need for innovative treatments for KOA. While physiotherapy is commonly used, its effectiveness alone is debated. Acupuncture shows promise, but the integration of computerized point- specific acupuncture with physiotherapy and its impact on KOA physical function is not well-explored (Atalay et al., 2021). This study aims to fill this gap by examining the combined impact of computerized point specific acupuncture and physiotherapy on KOA patients, providing insights for more effective rehabilitation.

This study employs a mixed-mode research design, utilizing quantitative and qualitative methodologies, which will be conducted in three phases. Phases 1 and 2, represent the quantitative component. In phase 1, 40 participants are randomly assigned to two groups: Group 1 (Comp-AcuPhysio: n = 20), participants will receive acupuncture treatment based on a computerized method of identifying point-specific acupuncture that generated by Acugraph system combined with personalized physiotherapy, while in Group 2 (Man-AcuPhysio: n = 20), participants in this group will receive acupuncture treatment based on the manual preferences of the acupuncturist and combined with personalized physiotherapy. Each group will undergo 60-minute sessions administered once a week for 12 weeks. Repeated measures of ANOVA will be utilized for data analysis to examine the effects of combining acupuncture with physiotherapeutic exercise over time. In Phase 2, the study extends to a 12-week follow-up (week 24) to investigate the long-term

effects of Comp-AcuPhysio compared to Man-AcuPhysio group. This phase involves all 40 participants from both groups, evaluating knee pain, physical function, and psychological well-being. Meanwhile, phase 3 focuses on the qualitative component and employs structured one-on-one interviews with participants to explore their psychological well-being, experiences, and perspectives, adding depth to the quantitative findings by examin the correlation between improvements in KOA physical function (assessed through well-established tools like KOOS and TUG) and the AcuGraph Personal Integrated Energetics (PIE) score. This study aims to fill gaps in existing research on KOA by thoroughly investigating the impact of Comp-AcuPhysio compared to Man-AcuPhysio group. The use of both qualitative and quantitative data aims to provide a detailed understanding of how this combined treatment affects KOA patients. The findings may have the potential to reshape how KOA is managed, offering new insights into rehabilitation practices.

The anticipated importance of the research findings from this comprehensive study on the combined effects of computerized point-specific acupuncture and physiotherapy for KOA is multi-faceted. The strengths of this proposed study, incorporating both quantitative and qualitative research approaches, contribute to a robust and nuanced understanding of the subject. Based on our knowledge, no study has been reported to examine the psychophysical effects of computerized point-specific acupuncture adjunct to physiotherapy compared to manual preferences acupuncture adjunct to physiotherapy in patients with KOA. Phase 1's significance lies in its ability to provide a thorough examination of the short-term effects of the combined intervention, offering immediate insights into its impact on knee pain, physical function, and psychological well-being. In addition, the correlation between improvements in KOA physical function and the AcuGraph PIE score is expected to unveil valuable insights into the interconnectedness of meridian energy systems and physical well-being. On the other hand, Phase 2 extends this inquiry, focusing on the long-term effects over a 12-week follow-up period, contributing valuable data on sustained improvements. The importance of Phase 3, with its qualitative exploration through structured interviews, adds depth and context to the overall findings by capturing patients' experiences and perspectives. Hence, the integration of computerized point specific acupuncture and physiotherapy, emphasizing holistic well-being and long-term effects, has the potential to redefine KOA rehabilitation practices, providing a comprehensive insight into treatment impact and patient experiences to enhance global management strategies.

1.2 Problem Statement

Existing KOA rehabilitation methods have proven insufficient in delivering optimal outcomes (Kamsan et al., 2020). Therefore, there is a pressing need to explore alternative treatments that integrate complementary medicine approaches. One such promising avenue is therapeutic acupuncture (Kwak et al., 2023). However, a significant challenge arises from the scarcity of information regarding the combined effects of point-specific acupuncture and therapeutic exercise, leaving the effectiveness of this integrated approach uncertain and demanding further investigation (Foster et al., 2007). There are several gaps in knowledge with specific reference to the application of acupuncture in managing KOA. For example, previous studies have focused on pain reduction and physical function improvement, neglecting equally essential aspects of psychological well-being and patients' subjective perceptions, which play a significant role in overall quality of life and treatment outcomes (Tian et al., 2022). Furthermore, previous studies have mainly investigated short-term effects, with limited knowledge of long-term outcomes (Jia et al., 2020). This is important considering KOA is a chronic disease that requires long-standing rehabilitation.

Moreover, no previous study has reported the correlation between improvements in physical function, as assessed by KOOS, TUG, and active knee flexion range, and the PIE (Personal Integrated Energetics) score, which reflects the overall health of the meridian energy system. This correlation can provide valuable insights into the potential link between traditional medical assessments and complementary energy-based health measures, potentially offering a more holistic approach to healthcare and enabling a more comprehensive understanding of patient well-being. Additionally, the choice of acupoints varies depending on the acupuncturist's preference in most research, leading to a lack of standardization that may influence observed results. Therefore, a comprehensive understanding. The mixed-mode research design seeks to bridge this gap and offer valuable insights for optimizing the treatment approach for individuals with KOA. By harnessing the potential benefits of acupuncture and physiotherapeutic exercise through a combination of quantitative and qualitative research methods, this study aims to provide a more comprehensive understanding of the subject.

1.3 Research Questions

1) What are the short- and long-term effects of computerized method of identifying point-specific acupuncture generated by Acugraph system as an adjunct to physiotherapeutic exercise compared to manual preferences acupuncture with physiotherapeutic exercise on knee pain, physical function, psychological well-being, and health perception among adults with knee osteoarthritis?
2) Is there a significant correlation between the improvements in physical function, as assessed by KOOS, TUG, and active knee flexion range, and the PIE (Personal Integrated Energetics) score, which reflects the overall health of the meridian energy system?

1.4 Research Objectives

1.4.1 General objectives:

1) To examine and compare the short- and long-term effects of computerized method of identifying point-specific acupuncture generated by Acugraph system as an adjunct to physiotherapeutic exercise, in contrast to manual preferences acupucnture with physiotherapeutic exercise, on knee pain, physical function, psychological well-being, and health perception among adults with knee osteoarthritis.
2) To determine whether a statistically significant correlation exists between improvements in physical function, as measured by KOOS, TUG, and active knee flexion range, and the PIE (Personal Integrated Energetics) score as an indicator of the overall health of the meridian energy system.

1.4.2 Specific objectives:

1) To examine the short-term effects (immediate following 12-weekly intervention) of computerized point-specific acupuncture as an adjunct to physiotherapeutic exercise compared to manual preferences acupuncture with physiotherapeutic exercise on pain, physical function, and psychological well-being among adults with KOA.
2) To examine the long-term effects (following a three-month follow-up) of computerized point-specific acupuncture as an adjunct to physiotherapeutic exercise compared to manual preferences acupuncture with physiotherapeutic exercise on pain, physical function, and psychological well-being among adults with KOA.

3) To explore qualitatively the experiences and perceptions of adults with KOA following a 12-week intervention of computerized point-specific acupuncture combined with physiotherapeutic exercise compared to manual preferences acupuncture with physiotherapeutic exercise.
4) To evaluate the relationship between changes in knee pain and physical function (as quantified by the Knee injury and Osteoarthritis Outcome Score, and the Timed Up and Go test) against the PIE score, which serves as an indicator of the overall health of the meridian energy system.

1.5 Research Hypothesis

(1) Adults with knee osteoarthritis (KOA) who receive computerized method of identifying point-specific acupuncture generated by Acugraph system in combination with physiotherapeutic exercise are expected to demonstrate significantly more significant improvements in knee pain, physical function, psychological well-being, and health perception when compared to those who undergo physiotherapeutic exercise alone.

(2) A significant positive correlation between improvements in physical function, as measured by the Knee injury and Osteoarthritis Outcome Score (KOOS), the Timed Up and Go (TUG) test, active knee flexion range, and the AcuGraph Personal Integrated Energetics (PIE) score. As physical function improves, a corresponding positive PIE score will indicate a connection between enhanced musculoskeletal well-being and the overall health of the meridian energy system.

1.6 Research Significance

The clinical implications of our research findings bear significant relevance to the field of KOA management, with potential benefits extending across individual patients, societal structures, and the healthcare system. Establishing the efficacy of a treatment protocol involving computerized method of identifying point-specific acupuncture generated by Acugraph system combined with physiotherapy holds promise for enhancing outcomes in KOA patients. This innovative approach can alleviate knee pain, enhance physical function, and improve psychological well-being and health perception. We opt for acupuncture in managing KOA because it offers a personalized and holistic approach. By addressing KOA's physical and psychological aspects, acupuncture tailors the treatment to each individual, providing a

comprehensive healing experience. Our study explores the relationship between improved knee function in KOA, as measured by tools like the AcuGraph Personal Integrated Energetics (PIE) score. This not only deepens our understanding of individual health but also empowers patients to play an active role in making decisions about their care.

Furthermore, enhancing the well-being of individuals with KOA could increase societal productivity. As patients regain the ability to participate actively in social and economic activities, the potential ripple effect on overall productivity becomes evident. Simultaneously, by reducing the burden of KOA-related disability, our research aims to play a role in cultivating a healthier and more active population. Our study could bring about significant changes within the healthcare system. If we prove that combining acupuncture and physiotherapy effectively treats knee osteoarthritis, it has the potential to be a recommended alternative approach.

1.7 Research Framework

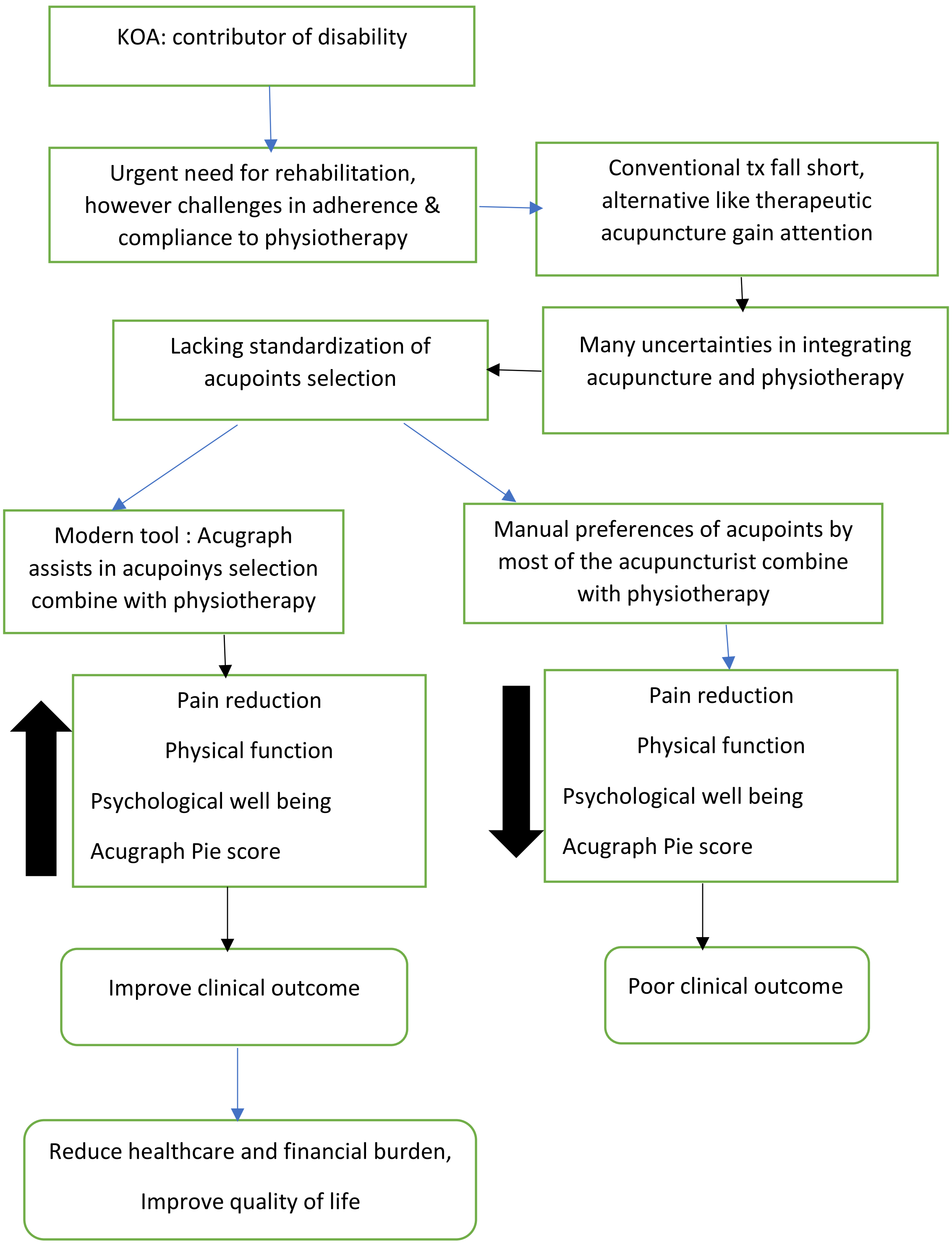

2.0 Literature Review

This proposal section will delve into the current body of literature surrounding the topic. The literature review will explore the pathophysiology of KOA, its prevalence, and the consequential effects of KOA on both physical and psychological well-being. A comprehensive examination of contemporary management strategies will lead to an in-depth analysis of acupuncture as an alternative therapeutic approach. The mechanism of acupuncture therapy will be dissected, followed by a thorough evaluation of clinical evidence concerning its impact on pain, physical function, and psychological well-being. The review will conclude by summarizing key findings and identifying existing gaps in knowledge, paving the way for future research endeavours.

2.1 Pathophysiology of KOA

Clinical investigations are actively exploring the elusive origins of KOA, with current findings indicating a lack of definitive understanding (Han et al., 2021). It is currently acknowledged as a form of panarthritis, affecting every aspect of a joint, including calcified cartilage, subchondral bone, capsular ligaments, and synovial fluid (Kaspiris et al., 2023). KOA may occur as a result of multiple factors. Mechanical factors that were engaging in repetitive activities, such as frequent kneeling, heavy lifting, and having weak knee extensor muscles, are identified as a crucial contributor to the prevalence of KOA, making joints more susceptible to damage (Primorac et al., 2020). The ongoing inflammatory response in KOA contributes to the deterioration of cartilage and other joint components, intensifying discomfort and functional limitations (Richebé et al., 2018). Characteristic symptoms of KOA include knee pain, restricted knee movement, joint stiffness, muscle weakness, functional mobility impairments, and postural balance disturbances (Hawker, 2019)

2.2 Prevalence and Impact of KOA

The global prevalence of KOA has continuously increased, with approximately 654 million individuals diagnosed with KOA in 2020 (Cui et al., 2020). It is estimated that by 2050, about 130 million individuals worldwide will experience OA, with 40 million of them facing severe disability (Mobasheri et al., 2019). OA was the 38th most impactful among 291 diseases, considering the overall burden measured by disability-adjusted life year (Johnson et al., 2021). It will become more

common in southeast Asia and other developing areas in the next few decades (Nguyen, 2014). In Malaysia, the prevalence rates for knee pain and self-reported knee osteoarthritis symptoms were 33.3% and 30.8%, respectively (Mat et al., 2019). Significantly, there were notable ethnic disparities, with Malays showing the highest crude prevalence of knee pain at 44.6%, followed by Indians at 31.9% and Chinese at 23.5% (Mat et al., 2019). As per the National Health and Morbidity Survey (NHMS) 2015, around 30.0% of adults in Malaysia were overweight, and 17.7% were obese. Because of these factors, Malaysians are likely to have a higher chance of experiencing more cases of OA (Figure 2.1) (Zamri et al., 2021).

Knee osteoarthritis (KOA) poses a complex challenge, requiring comprehensive attention and intervention due to its significant impact on individuals, healthcare systems, and the global economy. Studies stated that the rising prevalence of KOA places a substantial strain on the health system, and this burden is particularly evident in primary care settings, where most chronic conditions are managed (Xie et al., 2016) (Chen et al., 2012). Thus, the considerable financial burden of osteoarthritis (OA) within primary care significantly exacerbates the global economic challenges health systems encounter. A review emphasizes increased rates of primary care consultations and hospital admissions among those with KOA, coupled with a heightened risk of all-cause mortality compared to age- and sex-matched controls (Garin et al., 2016), demonstrated KOA emerges as a substantial cause of morbidity; this highlights the necessity of a comprehensive strategy for managing KOA, taking into account its effects on both primary care services and hospital admissions.

The widespread occurrence of knee osteoarthritis (KOA) globally from 2000 to 2020 adds to the overall burden (Cui et al., 2020). This prevalence results in significant and increasing financial pressure, with noteworthy contributions from direct costs like medications and hospitalization, which can be seen in a study by Leifer (2022), whereby indirect costs arising from work-related losses and home-care expenses further magnify the financial impact of KOA (Leifer et al., 2022). Beyond the physical manifestations, KOA has the potential to lead to enduring disabilities, impacting both physical and mental health (AHMAD et al., 2018). Addressing psychological symptoms emerges as a crucial aspect of KOA treatment, with individuals experiencing pain often exhibiting long-term depression (Rathbun et al., 2018). The information above highlights the interconnectedness of physical and mental well-being in the context of KOA.

2.3 Current Management of KOA

Due to its chronic degenerative nature, healthcare professionals frequently opt for a pharmaceutical strategy, typically involving nonsteroidal anti-inflammatory drugs (NSAIDs) and intra-articular injections, to mitigate pain and postpone the need for surgical intervention (Magni et al., 2021). However, extended pharmacological treatment often leads to side effects, such as peptic ulcers and gastrointestinal bleeding (Testa et al., 2021). The Osteoarthritis Research Society International (OARSI) and The Medical Journal of Australia (MJA) suggest non-drug treatments like exercise, education, and weight management to reduce KOA pain. The American College of Rheumatology (ACR) provides OA management recommendations, including acupuncture, for patients with chronic moderate to severe pain. However, the American Academy of Orthopaedic Surgeons (AAOS) doesn't recommend or oppose specific treatments for knee OA due to insufficient evidence and advises against acupuncture for the same reason (Rees, 2017).

In response to this concern, non-pharmacological approaches are gaining attention, and physiotherapeutic rehabilitation has been suggested as an alternative, per international KOA guidelines and supported by Kolasinski et al. (2022) and Bannuru et al. (2019). Despite the significant joint deterioration and functional limitations at mild to moderate knee OA, intervention is warranted (Bannuru et al., 2019; Kolasinski et al., 2020). However, it hasn't reached the stage where more invasive treatments, like surgery, are necessary. Improving muscle strength and joint stability at this stage can be achieved through strengthening exercises (Rizvi et al., 2023). However, exercise is limited because movement could induce more pain in KOA than rest (Zeng et al., 2021). The fear of pain in KOA often prevents them from flexing and stretching the knee joint, leading to the gradual development of joint mobility issues (Focht, 2006). Yet, a study found that doing resistance exercises for eight weeks significantly improved the abilities of people with KOA, such as function, strength, and mobility (Pazit et al., 2018). Using elastic bands for exercises, which are flexible and accessible, has been shown to make a big difference in the lower-body function of women with KOA after doing leg press exercises for eight weeks (Chang et al., 2012). However, it's not clear from current research whether using elastic bands for quadriceps femoris training is better at relieving pain than traditional exercises for KOA patients (León-Ballesteros et al., 2020).

Moreover, due to insufficient adherence and compliance with rehabilitation exercises, KOA patients fail to meet the recommended exercise levels; this leads to suboptimal improvements

in clinical outcomes (L. Wang et al., 2020). Although there's plenty of evidence showing that exercise is helpful for KOA, current management is not enough to produce optimal outcomes; thus, there is a need for alternative treatment as an adjunct to complement the current management.

2.4 Acupuncture as An Alternative Management in Musculoskeletal Disorder

Alongside physiotherapeutic interventions, complementary therapies have been incorporated to enhance clinical outcomes, with acupuncture being a more recent and broadly accepted practice, particularly in the Southeast Asian region (MacPherson & Hammerschlag, 2012). Acupuncture, with a history spanning over 3,000 years and originating in China, has been recognized for its potential to manage chronic musculoskeletal pain associated with conditions like osteoarthritis, rheumatoid arthritis, fibromyalgia, and chronic low back pain (Zhang & Wang, 2020), it is now endorsed as a treatment for 77 conditions, including 16 linked to inflammatory pain (Zhang et al., 2023). Moreover, recent studies by Zhang et al. (2020), Liu et al. (2022), and Li et al. (2019) have contributed to the growing body of evidence supporting acupuncture as a therapeutic modality for alleviating pain in musculoskeletal conditions (Li et al., 2019; C.-Y. Liu et al., 2022; Zhang et al., 2020). The findings highlight acupuncture's potential as a valuable component in the spectrum of treatments for managing musculoskeletal pain, warranting further exploration and consideration in comprehensive pain management strategies.

Acupuncture, a traditional Chinese medicine technique, involves the insertion of thin sterile needles into specific points on the body. It is rooted in the concept of Qi energy flow through meridians, with the theory suggesting that imbalances or blockages in Qi can lead to pain and illness. By stimulating the acupuncture points, the balance of Qi can be restored, promoting healing and pain relief (Thompson, 2017). Acupuncture is thought to trigger the release of endorphins, natural pain-relieving chemicals, and modulate the nervous system to reduce pain signals and induce relaxation (Miao et al., 2023). It is believed that emphasising specific acupoints is a holistic approach to addressing disease symptoms and underlying causes (Zhang & Zhu, 2023).

2.5 Clinical Evidence of Acupuncture Therapy In KOA

The clinical effects of acupuncture have been widely investigated, with relevant articles summarizing the effects as presented in Table 2.1.

2.5.1 Effects of Acupuncture on Pain

The World Health Organization (WHO) has recognized the benefits of acupuncture for various conditions, explicitly citing knee pain as a confirmed treatment based on over 100 clinical trials (Rees, 2017). Acupuncture is considered a safe alternative for KOA management with positive effects and low associated risks (Khan et al., 2018; White et al., 2001). In contrast, commonly recommended intra-articular injections for KOA management, as proposed by Cooper et al. (2017) (Cooper et al., 2017), carry a risk of developing septic arthritis from intra-articular steroid injections (Testa et al., 2021). Acupuncture is relatively safe, with minimal risks of organ or tissue injuries, infections, local adverse effects, or reactions, including dizziness or syncope (Bäumler et al., 2021; Chan et al., 2017).

A recent trial suggests that both electroacupuncture and manual acupuncture are effective and safe for KOA (R. Li et al., 2020), while another study by Li et al. (2023) concluded electroacupuncture has a similar effect as analgesics in terms of pain reduction and joint function on KOA (P. Li et al., 2023). However, compared to physiotherapy, neither acupuncture method significantly differs in pain reduction and physical function (Atalay et al., 2021). Given the significant improvement observed with the combination of acupuncture and pharmacological treatment compared to pharmacological treatment alone (Lele et al., 2019), further research is warranted to explore the effects of acupuncture as an adjunct to physiotherapeutic exercise in KOA management.

2.5.2 Effects of Acupuncture on Physical Function

A recent study by Zhou et al. 2023 proposes that maintaining spleen and stomach health in Traditional Chinese Medicine (TCM) is crucial for resisting meridian obstruction (Zhou et al., 2023). Applying acupuncture to foot acupoints related to the spleen and stomach meridian, such as Sanyinjiao and Zusanli, may improve physical function in KOA (Zhou et al., 2023). However, the long-term prognosis and application of spleen and stomach acupoints remain unclear. Contrary to this, numerous high-quality studies, including one by Tian et al. (2022), have indicated that acupuncture exhibits short-term efficacy in managing KOA (Tian et al., 2022). A meta-analysis conducted by Lin et al. (2016), which analysed 10 trials trials published through March 2015 concluded that acupuncture has short and long-term effect on physical function after 13 and 26 weeks (Lin et al., 2016). In contra with study by Tian et al (2022), meta-analysis by Lin et al

(2016) demonstrates that acupuncture can improve long-term physical function in patients with chronic knee pain. The lack of clarity on the duration of these effects and whether they are sustained beyond the specified timeframes limits the practical implications of the findings. Moreover, although several studies with large sample sizes demonstrate that acupuncture effectively improves physical function in KOA, the comparison in this study was limited to a placebo group, excluding consideration of active treatments (Tian et al., 2022). Consequently, these findings may not be universally applicable to all KOA patients.

2.5.3 Effects of Acupuncture on Psychological Well-Being

Most studies indicate that acupuncture can notably enhance emotional symptoms such as (Jiang et al., 2022; Wang et al., 2022), migraine (Zhang et al., 2021), chronic pain patient's psychological disorders such as anxiety and depression (Lin et al., 2021). A recent systematic review of high-quality trials indicated acupuncture positively influences KOA pain reduction; however, its effects on quality of life is inconsistent (Tian et al., 2022). Specifically, psychological and neurobiological factors have consistently been linked to knee OA symptoms (Johnson et al., 2021); however, the links between emotional well-being and pain processing in knee osteoarthritis are not fully comprehended. It is worth noting that most studies have primarily reported the short-term effects of psychological well-being (Jia et al., 2020; Tian et al., 2022), lacking a qualitative approach to delve into this context, thereby indicating a need for further investigation.

2.5.4 Safety and Adverse Effects of Acupuncture

A recent systematic review by Tian et al. (2022) indicated that acupuncture is an effective alternative therapy for patients with KOA, especially those experiencing long-term pain; however, this study included individuals with mild, moderate, and advanced KOA, creating challenges in drawing definitive conclusions about acupuncture's effectiveness and safety for specific patient subgroups (Tian et al., 2022). Although possible adverse effects that arise from acupuncture include local hematoma, tiredness, weakness, drowsiness, nausea, and headache (R. Li et al., 2023), a recent bibliometric approach and systematic review demonstrated acupuncture is effective in pain reduction and safe with minimal risk and adverse effects (Vickers et al., 2012; Xu et al., 2023). In addition, several previous studies on the safety of acupuncture reported that the treatment is safe in managing patients with cancer care (Lu et al., 2008), neurological disorders (Ye et al., 2023),

Post-traumatic stress disorder (PTSD) (Ding et al., 2020). Furthermore, an overview of a systematic review by Chan et al. (2017), which analysed 17 articles, found that serious adverse effects from acupuncture are rare, but there is a need to consider acupuncturist's training credibility, and patient safety should be a core part of acupuncture education (Chan et al., 2017).

2.6 AcuGraph in Acupunture Application

Acupuncture points are specific locations on the body's surface connected to energy pathways, known as meridians, in Traditional Chinese Medicine. Practitioners use these points to insert needles and influence the body's energy flow, referred to as "qi." (Langevin & Wayne, 2018). Physiologically, acupuncture points are often found in cutaneous regions characterized by low electrical impedance and high electrical conductivity, with increased blood flow, more capillaries, and distinct connective tissue structure (Kim et al., 2020; Lim et al., 2018). Despite the historical theory of acupuncture in ancient years (Y.-X. Li et al., 2023), the lack of standardization and consistent guidelines complicates the determination of acupuncture treatment techniques; specifically, the selection of acupoints lacks uniformity (Mi et al., 2023). Recent randomized controlled trials have generated conflicting results regarding the appointment of acupuncture points (Tang et al., 2018). While some studies support sensitized acupoints, limited evidence supports this hypothesis. These trials suggested that highly sensitized points potentially benefit patients as much as less sensitized points (Tang et al., 2018). In contrast, other studies reached the opposite conclusion, suggesting that selecting acupoints with lower versus higher pain thresholds both effectively treat KOA (J. Liu et al., 2022). These contrasting results underscore the need to explore acupuncture therapies that are more standardized and readily accepted by KOA patients.

In modern acupuncture, tools like AcuGraph are employed to assess and quantify the electrical properties of these acupuncture points. AcuGraph uses a device to measure electrical impedance at various points, creating graphical representations that help acupuncturists visualize and analyze the balance and vitality of meridians. Lower impedance readings may suggest better energy flow, guiding the selection of acupuncture points for interventions(Mist et al., 2011). While AcuGraph enhances diagnostic capabilities by providing objective measurements, its limited usage in studies hampers the integration of acupuncture practices with modern technology. Hence, it is essential to undertake research using AcuGraph, connecting traditional acupuncture principles with modern technology and propelling the field forward.

2.7 Summary of Literature Findings

Despite a growing body of evidence supporting acupuncture for knee osteoarthritis (KOA), it's not yet considered a standard treatment due to limited high-quality clinical evidence. Issues like small sample sizes and lack of standardization hinder conclusive results, as seen in Lin et al (2018) studies. A study by Foster et al. (2007) failed to demonstrate the additional acupuncture on exercise approach beneficial to knee OA patients partly due to the absence of a placebo group, lack of blinding and small sample size (Foster et al., 2007). The combined application of acupuncture with exercise therapy remains unclear regarding its superiority over exercise therapy alone. The lack of standardization in acupuncture treatment techniques and acupoint selection complicates interpretation, leading to intervention variations across studies. Recent trials show conflicting results on sensitized acupoints, emphasizing the need for more standardized acupuncture therapies (J. Liu et al., 2022; Tang et al., 2018).

These contrasting results underscore the need to explore acupuncture therapies that are more standardized and readily accepted by KOA patients. While the short-term effects of acupuncture on KOA are acknowledged, long-term effects remain controversial due to methodological issues (Jia et al., 2020). This information scarcity suggests that the current evidence on acupuncture is lacking, primarily due to methodological problems, necessitating further investigation to clarify the long-term effects. The research on acupuncture deserves further investigation, as the re-analyzed results from (Zhou et al., 2016) concerning Hinman et al.'s (Hinman et al., 2014) study successfully identified issues in the previous research. The conclusions drawn from Hinman's study indicate the effectiveness of acupuncture, indicating the need for supportive evidence in current clinical studies. Thus, recognizing this knowledge gap, our proposed study aims to examine the short- and long-term on the effects of computerized point-specific acupuncture that determined by Acugraph with physiotherapy as compare to manual preferences acupuncture with physotherapeutic exercise on knee pain, physical function, and disability levels among adults with mild to moderate KOA.

Table 2.1. Clinical evidence on the effects of acupuncture in knee osteoarthritis.

No	Study	Methodology	Findings	Comments
1.	(J. Liu et al., 2022)<br><br>Clinical trial<br><br>Effects of acupuncture at acupoints with lower versus higher pain threshold for knee osteoarthritis: a multicenter randomized controlled trial	• Study design: RCT<br>• Sample size: 666 patients<br>LPT = 222<br>HPT = 222<br>• Intervention: Patients were randomly assigned to receive acupuncture at acupoints with either lower or higher pressure thresholds (LPT and HPT groups), or no acupuncture (waiting-list group).<br>• Intervention frequency and duration: 16 weeks<br>• Outcomes: WOMAC, SF-12 score and active knee range of motion.	• Both the LPT and HPT groups had similar effects in reducing WOMAC total scores over 16 weeks.<br>• Both LPT (−9.77) and HPT (−11.97) groups demonstrated greater reductions in WOMAC total scores compared to the waiting-list group.<br>• No significant differences in SF-12 scores and ROM between the LPT and HPT groups.	• Potential Bias in Acupoint Selection: The study divided patients into groups based on PT for acupoint selection. The determination of PT can be influenced by various factors, including the individual's pain perception and psychological state. This subjectivity in PT measurement could introduce bias into the study's design.<br>• No Comparative Non-Acupuncture Group: While the study compared two acupuncture groups (LPT and HPT) to a waiting-list group, it would have been beneficial to include a non-acupuncture, standard care group for a more comprehensive comparison

2.	(Jia et al., 2020)<br><br>Clinical trials<br><br>Acupuncture for knee osteoarthritis with sensitized acupoints: results from a pilot, feasibility randomized controlled trial	• Study design: RCT<br>• Sample size: 36<br>HS group: 18<br>NS group: 18<br>• Intervention: Patients were randomly assigned to receive acupuncture at acupoints HS and NS<br>• Intervention frequency and duration: 3 sessions for 4 weeks, follow up at 4,8,12, 16 weeks.<br>• Outcomes: Retention rates, WOMAC scores, SF-12 scores	• Acceptable retention rates: 77.8%<br>• Secondary outcomes suggested that acupuncture on the highly sensitized points may help to achieve improved therapeutic effects.	• the study offers valuable insights into the short-term effects of intensive acupuncture for knee OA, but does not provide details on the duration and timing of the follow-up assessments.
3.	(Tian et al., 2022)<br><br>Meta- analysis<br><br>Acupuncture for Knee Osteoarthritis: A Systematic Review	• Searched multiple databases and sources for RCTs on acupuncture for KOA from inception to December 2021.<br>• Meta-Analysis: Conducted a meta-analysis using	• Acupuncture had a positive effect on reducing pain and improving patients' functional activities.<br>• Did not significantly relieve patient stiffness	• Short Duration: The treatment lasted only 4 weeks with a 16-week follow-up, which might not be enough to grasp its long-term impact on a chronic condition like knee osteoarthritis.

	of Randomized Clinical Trials with Meta-Analyses and Trial Sequential Analyses	Cochrane systematic review methods and Review Manager 5.4 and Trial Sequential Analysis (TSA) to assess the quality of the evidence.<br>• Inclusion of Studies: Included eleven RCTs with a total of 2484 KOA patients that met the inclusion criteria for the meta-analysis.	or demonstrate differences in improving mental and physical health quality of life compared to sham acupuncture.<br>• Trial Sequential Analysis: Utilized TSA to control random errors and determine the required information size, indicating the need for more RCTs in future studies.	• Lack of Control Group: The study faced a limitation in not having a control group, creating difficulty in establishing whether the effects observed were solely attributed to acupuncture or influenced by other factors.<br>• Lack of Detailed Clinical Outcomes: The study lacked detailed clinical outcomes, such as changes in pain, function, or quality of life, impeding a thorough assessment of acupuncture's potential benefits.
4.	(Foster et al., 2007)<br><br>Clinical trial<br><br>Acupuncture as an adjunct to exercise based physiotherapy for osteoarthritis of	• Study design: RCT<br>• Sample size: 352 patients.<br>• Intervention: Patient randomly assigned to: Advice and exercise (n=116), advice and exercise plus true acupuncture (n=117), and	• No significant differences between advice and exercise alone and the acupuncture groups, penetrating acupuncture. Similar non-significant differences were	• Additional research is imperative to achieve a more profound comprehension of the enhancements in quality of life and to comprehensively explore the factors influencing these improvements.

	the knee: randomised controlled trial.	advice and exercise plus non-penetrating acupuncture (n=119).<br>• Outcome: Western Ontario and McMaster Universities osteoarthritis index, function, pain intensity	observed at other follow-up time points.	• The article exhibits a deficiency in specific details pertaining to inclusion and exclusion criteria, risk of bias assessment for the included studies, and the quality of acupuncture interventions. This suggests the need for further clinical studies in the future to address these gaps.
5	(Tang et al., 2018)<br><br>Clinical trial<br><br>Acupuncture treatment for knee osteoarthritis with sensitive points: protocol for a multicentre randomised controlled trial	• Study design: RCT<br>• Sample size: 666 patienst<br>• HS: 222<br>• LS: 222<br>• No acupuncture:222<br>• Participants will be randomly assigned to one of three groups: high-sensitisation group, low/non-sensitisation group, or waiting-list group/ no acupuncture	• acupuncture at sensitive points leads to a better therapeutic effect<br>• provide compelling evidence on the selection of acupuncture points to achieve improved KOA outcomes.<br>• the result findings may change the guideline	• Generalizability: The study was conducted in the Midlands, United Kingdom, which may limit the generalizability of the findings to other populations or geographic regions. Cultural and healthcare system differences can affect how patients respond to treatments, including acupuncture.<br>• Potential bias: The study did not include blinding for patients, physiotherapists, or assessors. This lack of blinding could introduce

		• Outcome measure: WOMAC, SF 12, Knee ROM	• for practising acupuncture on KOA	bias into the study, as participants and those administering the interventions or assessing outcomes may have had expectations or preferences that could influence the results.

RCT: Randomized Controlled trial; LPT: High Pain Threshold; LPT: Low Pain Threshold; WOMAC: Western Ontario and McMaster Universities Osteoarthritis Index; ROM: Range of motion; SF 12: 12-Item Short Form Health Survey; HS: High sensitization; LS: Low Sensitization

3.0 Methodology

The proposed study will consist of three phases:

- Phase 1: quantitative design - randomized controlled trial (short-term outcomes)
- Phase 2: quantitative design - randomized controlled trial (long-term outcomes)
- Phase 3: qualitative study - structured one-on-one interviews (participants' experiences and perspectives)

3.1 Phase 1: Quantitative Design - Randomized Controlled Trial (Short-Term Outcomes)

3.1.1 Study Design

This study will be a parallel group, randomized controlled trial. The study protocol was designed based on the CONSORT guidelines for clinical trial. Participants will be randomly assigned to one of two groups: computerized point-specific acupuncture that determined by Acugraph with physiotherapy (Comp-AcuPhysio) and manual preferences acupuncture with physotherapeutic exercise (Man-AcuPhysio).

3.1.2 Study Settings

This study will be conducted at Grand Care Rehab Seremban, a private physiotherapy center located in Negeri Sembilan, Malaysia. Grand Care Rehab offers conventional physiotherapy rehabilitation services, including pain management, sports injury treatment, stroke rehabilitation, and scoliosis rehabilitation. Additionally, the center specializes in complementary medicine approaches, particularly traditional Chinese medicine, including acupuncture.

3.1.3 Target Population

The target population for this study will encompass patients with mild to moderate knee osteoarthritis (KOA), as classified by the Kellgren Lawrence classification in grade 2 and grade 3 , who have been referred by their physicians.

Kellgren Lawrence classification: grade 2 (minimal): definite osteophytes and possible joint space narrowing; grade 3 (moderate): moderate multiple osteophytes, definite narrowing of joint space and some sclerosis and possible deformity of bone ends

The inclusion and exclusion criteria are as follows:

3.1.3.1 Inclusion criteria

- Adults aged 18 years and above of both sexes.
- Diagnosed with unilateral or bilateral KOA by an orthopedic doctor.
- Radiologic confirmation of KOA grade 2 or 3 OA in knee joints according to Kellgren-Lawrence, X ray that performed less than 12 months.

3.1.3.2 Exclusion criteria

- Patients who are unable to comply with the study intervention.
- Patients with a history of trauma, ligament damage, fracture, or surgery on the knee(s) within the past 6 months, causing pain or functional problems
- Patients with a history of local tumor/malignancy at the knee.
- Patients with a history of prolotherapy, hyaluronic acid injections, or corticosteroid injections within the past 6 months.
- Patients who are oversensitive to needles.

3.1.4 Sample Size Calculation

Sample size determination was performed using G*Power software version 3.1.9.7. The calculation relied on a minimum clinically significant difference (MCID) of 11 points for the primary outcome, which was assessed using the Knee Injury and Osteoarthritis Outcome Score (KOOS). This estimation considered a predetermined power of 90%, an effect size of 0.3, a significance level of 5%, and an anticipated dropout rate of 30%. Consequently, the calculated total sample size was 40 participants, evenly divided into two groups of 20 participants each.

3.1.5 Sampling and randomization

In the proposed study, eligible participants will be screened and recruited from Grand Care Rehab Seremban, a private physiotherapy center located in Negeri Sembilan, Malaysia. Patients with mild to moderate KOA will be screened by a research assistant who is not involved in the intervention or outcome assessment, based on the criteria mentioned earlier. Upon meeting the inclusion criteria, eligible participants (n = 40) will be required to provide signed informed consent and will be given a research information sheet. They will then be randomly allocated to one of two groups: the Comp-AcuPhysio (n = 20) or Man-AcuPhysio (n = 20) using a computerized simple block randomization

method. The randomization process will be conducted by the principal investigator, who is not involved in the intervention or outcome assessment.

3.1.6 Group allocation and intervention

In this study, participants will be randomly allocated to one of two groups:

- Group 1 (Comp-AcuPhysio, n=20):), participants in this group will receive acupuncture treatment (approximately 20 minutes) based on a computerized method of identifying point-specific acupuncture that generated by Acugraph system and combined with personalized physiotherapy (approximately 40 minutes) as the intervention, totaling 60 minutes per session. This sequence of intervention is random and interventions will be administered once a week for 12 consecutive weeks.

  Group 2 (Man-AcuPhysio, n=20): participants in this group will receive acupuncture treatment (approxiamately 20 minutes) based on the manual preferences of the acupuncturist and combined with personalized physiotherapy (approximately 40 minutes) as the intervention, lasting for 60 minutes per session. However, for blinding participants' purposes, placebo Acugragh analysis will be conducted to blind the group allocation. The treatment will be carried out for 12 sessions, once a week for 12 consecutive weeks.

The acupuncture-related AcuGragh evaluation for both groups will be carried out by a single acupuncturist who is not involved in prescribing the intervention. Meanwhile, for standardization purposes, interventions for both groups will be conducted for 12 sessions, once a week for 12 consecutive weeks. Treatment (acupuncture and physiotherapy) for both groups (Comp-AcuPhysio and Man-AcuPhysio) will be conducted by a single qualified physiotherapist cum acupuncturist who is not involved in the outcome assessment.

The protocols for physiotherapeutic exercises (Allen et al., 2019; Chao et al., 2021; Foster et al., 2023; Holden et al., 2023; Zeng et al., 2021) and acupuncture (Li et al., 2019; Tian et al., 2022; Z. Wang et al., 2020; Wu et al., 2020; Zhang et al., 2020), treatments have been adapted from previous relevant studies (Atalay et al., 2021; Mamipour et al., 2023; Williamson et al., 2007), and are based on the recommended KOA guidelines (Bannuru et al., 2019; Fernandes et al., 2013; Kolasinski et al., 2020). Additionally, the acupuncture treatment protocol will be reviewed by an expert practitioner.

The details of the components of the physiotherapeutic exercises and acupuncture treatment are as follows:

a) *Physiotherapeutic exercise:*

The physiotherapeutic exercise will be prescribed as a supervised session. The components of the exercise will be adopted from previous studies that have been proven to produce favorable outcomes in KOA management (Ahmad et al., 2023;Gay et al, 2016; Suzuki et al, 2019; Button et a, 2015). The exercise prescription and progression will be modified accordingly based on the individual patient's KOA. Examples of the exercise components are as follows:

- Range of motion:
  Prone knee bend (2 sets, 10 repetitions each)
  Supine alternate knee bend (2 sets, 10 repetitions each)
- Stretching:
  Standing quadriceps stretch (1 set, 3-5 repetitions, 15-second hold each)
  Calf stretch in long sitting (1 set, 3-5 repetitions, 15-second hold each)
  Supine hamstring stretch (1 set, 3-5 repetitions, 15-second hold each)
- Strengthening:
  Sitting knee extension (Week 1-2: 1 set, 5 repetitions, 5-second hold; Week 3-5: 1 set, 7-10 repetitions, 5-10-second hold; Week 6-8: 2 sets, 10 repetitions, 10-second hold)
  Supine straight leg raise (Week 1-2: 1 set, 5 repetitions, 5-second hold; Week 3-5: 1 set, 7-10 repetitions, 5-10-second hold; Week 6-8: 2 sets, 10 repetitions, 10-second hold)
  Static quadriceps (Week 1-2: 1 set, 5 repetitions, 5-second hold; Week 3-5: 1 set, 7-10 repetitions, 5-10-second hold; Week 6-8: 2 sets, 10 repetitions, 10-second hold)
  Side-lying straight leg raise (Week 1-2: 1 set, 5 repetitions, 5-second hold; Week 3-5: 1 set, 7-10 repetitions, 5-10-second hold; Week 6-8: 2 sets, 10 repetitions, 10-second hold)
- Functional training:
  Sitting to stand (10 repetitions)
  Standing mini-squat (10 repetitions)
  Walking exercise (approximately 2 minutes for each item)

b) *Acupunture Treatment:*

In this study, the acupuncture treatment will be administered by a physiotherapist who has undergone specialized training, also referred to as an acupuncturist.

For Comp-AcuPhysio group, the process can be divided into two steps: first, the screening of treatment points using AcuGraph, and the second, the application of acupuncture treatment. Initially, KOA patients will be screened using The Acugraph system, which serves as an electrodermal screening device. It assesses energy flow and stress levels by measuring electrical conductance at specific acupuncture points on the skin. Analyzing these readings provides insights into overall health and meridian imbalances, playing a pivotal role in locating precise acupuncture points on the body. This ensures accurate and effective needle placement and ultimately enhances the precision of acupuncture treatments to optimize therapeutic outcomes by targeting the body's vital energy pathways. The monitoring computer records readings at specified points in a standard sequence, specifically at the Jing-Well acupuncture points on the fingers and toes, which are related to main organs: Lung (LU), pericardium (PC), heart (HT), small intestine (SI), triple energizer (TE), large intestine (LI), spleen (SP), liver (LR), kidney (KI), bladder (BL), gallbladder (GB), and stomach (ST). Acupoints specific to each participant will be generated by the computer to guide the specific point of treatment.

For Man-AcuPhysio group: Placbo Acugraph analysis will be conducted. The acupoints (Figure 3) used in the trial include Yanglingquan (GB34), Yinlingquan (SP9), Dubi (ST35), and Neixiyan (EX-LE4). At the same time, depending on the patient's condition, 2 local acupuncture points can be added follow acupuncturist preferences. Later, acupuncture treatment will be carried out. The acupuncturist performs a thorough assessment of the patient's condition, considering any contraindications and ensuring a sterile environment. In the next step, specific acupuncture points related to knee pain and arthritis will be targeted during the treatment. The location of these acupoints can vary based on the pathway of Qi flow. They may include points (1) close to the knee joint, particularly distal and proximal points around the antero-medial and antero-lateral parts of the knee joint, and (2) acupoints that are located away from the knee joints, such as those on the stomach and spleen meridian channels that pass through the knee area.

On average, the number of commonly applied acupoints in a typical acupuncture session for KOA may involve the use of approximately 4 to 12 acupuncture points in both group, these acupoints are carefully selected. Sterile, disposable needles are inserted into these points at the appropriate depth

(0.30: 25mm, or 0.30: 40mm), and 0.8 to 3cm was used as penetration depth for 20 minutes, using a gentle technique to minimize discomfort. The acupuncturist monitors the patient for any adverse reactions and adjusts needle placement or stimulation as needed. This treatment aims to alleviate pain, improve joint function, and promote overall well-being. Strict safety protocols, including infection control and patient comfort, are followed throughout the procedure

3.1.7 Outcomes Measures

In this phase, outcome measures will be assessed at two different intervals:

1. Baseline (pre-intervention): These measurements are conducted before the intervention to establish the initial state of the participants' KOA condition.
2. Immediately post-completion of intervention (short term, week-12): These measurements are taken immediately after the last intervention, which occurs at week 12.

All outcomes will be assessed by a blinded assessor who is not involved in carrying out the intervention. In addition to the outcome assessments, participants will be screened for potential adverse events, such as bruising, fatigue, weakness, and swelling, during each follow-up visit throughout the treatment. The list of outcomes to be measured in the study is as follows:

a) *Knee Injury and Osteoarthritis Outcome Score (KOOS):* The KOOS is a validated questionnaire used to assess the outcomes and subjective experiences of individuals with knee injuries or osteoarthritis. It measures various domains, including pain, symptoms, activities of daily living, sports and recreation, and quality of life. This provides a comprehensive evaluation of knee-related health status and aids in treatment assessment and research. KOOS demonstrates high reliability and validity, even in different languages. The test-retest reliability was excellent, with intraclass correlation coefficients ranging from 0.91 to 0.99 (Phatama, 2021).

b) *Active knee flexion ROM:* To measure knee flexion, line the stationary arm of the goniometer up with the greater trochanter along the outer thigh and the other arm of the goniometer up with the lateral malleolus of the ankle. The goniometer is easy to operate by hand and has a high reliability value, with an intraclass correlation coefficient of 0.997 for knee flexion in subjects with knee impairments (Remigio et al, 2017). Moreover, the goniometer is reliable for evaluating the range of knee flexion in different body positions, including supine, prone, and sitting positions, with

results highly correlated (r > 0.80) (Unver et al, 2009).

c) *TUG test:* The timed up and go test is used to assess functional mobility. It measures the time required to stand from a chair, walk 3 meters, and sit again in seconds. The average of two trials is recorded. The minimal detectable change for this test was reported as 1.10 seconds. The TUG test is used for this clinical trial due to its high reliability and validity for Grade 1-3 KOA (Sarac et al, 2022). The TUG test is recommended by the Osteoarthritis Research Society International (OARSI) for knee OA patients, with intra-rater and inter-rater reliability values of 0.97 (95% confidence interval [CI], 0.95 - 0.98) and 0.96 (95% confidence interval [CI], 0.94 - 0.97), respectively (Alghadir et al, 2015).

d) *The WHO-5 Well-Being Index:*The WHO-5 Well-Being Index: Developed by the World Health Organization, this index assesses overall psychological well-being by asking questions related to mood, vitality, and general well-being. The WHO-5 demonstrated strong internal consistency, with a Cronbach's alpha coefficient of 0.858 (Omani et al, 2019). It exhibited significant correlations with the PHQ-9 (r = -0.522), HADS-anxiety (r = -0.524), and HADS-depression (r = -0.630), thereby affirming its convergent validity (Omani et al, 2019).

e) *The Short Form 36 Health Survey (SF-36):* The SF36 is indeed a widely used questionnaire in health science research, but it is primarily designed to assess health-related quality of life rather than psychological well-being. However, because psychological well-being is a crucial component of overall health and quality of life, the SF-36 can provide valuable insights into a person's psychological state. The SF-36 is a responsive tool with total score SF-36 scores demonstrated the greatest effect sizes (> 0.9) (Clement et al, 2022). Scores of ≤52 in the MH subscale and ≤56 in the SF-36 effectively detected anxiety and depression, with good accuracy (Matcham et al, 2016). Similarly, MCS scores of ≤35 and ≤40 proved reliable for identifying anxiety and depression, with an additional threshold of ≤38 for detecting either condition (Matcham et al, 2016).

f) *Acugraph system:* This system is used to measure and analyze the energetic statuses of acupuncture meridians. Within the P.I.E. chart, each wedge of the circle represents one meridian

pathway. Those shown in green are balanced and functioning properly. The P.I.E. (Personal Integrated Energetics) score includes a complete analysis of all meridian pathways and reflects the overall health of your meridian energy system. A perfect score of 100 reflects complete balance and unimpeded flow of your Qi energy. A lower score indicates various imbalances that need to be addressed (Nagila et al, 2013)

3.1.8 Data Analysis

To examine differences between the treatment and control groups over time, a repeated measures ANOVA will be conducted. This analysis will allow us to assess the main effects of time, treatment group, and the interaction between time and treatment group. Additionally, effect sizes will be calculated using Cohen's d to determine the magnitude of the observed treatment effects. These analyses will provide valuable insights into the effectiveness of computerize points specific acupuncture as an adjunct to physiotherapeutic exercise in managing pain and improving physical function in individuals with knee osteoarthritis.

3.2 Phase 2: Quantitative Design - Randomized Controlled Trial (Long-Term Outcomes)

The second phase of this study aims to examine the long-term effects of Comp-AcuPhysio in comparison to Man-AcuPhysio on knee pain, physical function, and psychological well-being. This phase serves as a continuation of the first phase. During the second phase, outcome measures will be assessed at the week 24 follow-up, which occurs three months after the last treatment session and involves all 40 participants from both groups. All assessments will be conducted by a blinded assessor who has not been involved in delivering the intervention. The list of outcomes to be measured in the study includes the following: Knee Injury and Osteoarthritis Outcome Score (KOOS), active knee flexion range of motion (ROM), the timed up and go test, The WHO-5 Well-Being Index & Short Form 36 Health Survey (SF-36) and Acugraph system (PIE Score) evaluation.

Data collected will be analyzed using a two-way repeated measures ANOVA to examine differences between the treatment and control groups over time. This analysis will allow us to assess the main effects of time, treatment group, and the interaction between time and treatment group. Additionally, this study will investigate the relationship between changes in conventional physical function assessments (including KOOS, TUG, and active knee flexion range) and the meridian changes recorded by the Acugraph system. Specifically, we aim to determine if improvements in

physical function correlate with the energetic statuses of acupuncture meridians, with a particular focus on the P.I.E. (Personal Integrated Energetics) score. The P.I.E. score serves as a reflection of the overall health of the meridian energy system. This analysis will provide insights into whether changes in physical function align with the energetic dynamics of acupuncture meridians, shedding light on potential connections between these two aspects.

3.3 Phase 3: Qualitative Study - Structured One-On-One Interviews (Participants' Psychological Well-being, Experiences, And Perspectives)

In the third phase of this study, our objective is to qualitatively explore the experiences and perspectives of adults with knee osteoarthritis (KOA) who have undergone a 12-week intervention involving computerized point-specific acupuncture that determined by Acugraph system paired with physiotherapy and manual preferences acupucnture with physiotherapeutic exercise.

3.3.1 Procedures

To achieve this objective, this study will conduct individual interviews either in person or online, employing a semi structured interview method instead of structured interview. Structured interviews employ standardized and closed-ended questions, limiting flexibility in data collection, making them unsuitable for this study's needs (Gill et al., 2008). The use of semi-structured interviews in Phase 3 of the qualitative study design offers a flexible and tailored approach to data collection, fostering in-depth exploration of participants' perspectives and experiences while allowing for iterative questioning to uncover emergent themes (Gill & Baillie, 2018). This will involve seeking feedback and insights from research team to ensure the interview questions are appropriately designed to capture the nuanced aspects of participants' experiences and perspectives. Additionally, a pilot testing will be conducted to assess the clarity, relevance, and effectiveness of the questions in eliciting comprehensive responses from participants. By continuously refining and revising, we will ensure that our chosen interview questions are robust and capable of yielding pertinent insights relevant to our research topic.

We will arrange suitable physical or online meeting locations, ensuring that participants are comfortable with their chosen method. The interviews will be conducted one-on-one with the selected participants, with each interview being audio or video-recorded following the participants' consent. The interviews will be administered by a trained interviewer who is well-versed in the study's objectives but will maintain neutrality to prevent bias. During the semi-structured interviews, the

interviewer will initiate the conversation with predetermined research questions, allowing participants to elaborate on their experiences and perspectives. The interviewer also take comprehensive notes during the interviews, capturing both verbal responses and non-verbal cues and emotions.

The semi structured interview questions will be open-ended and are detailed as follows:

1. KOA symptoms perception: Can you describe if there are any changes in terms of knee pain or other KOA symptoms following the 12 sessions of treatment?
2. Knee function perception: Can you describe any changes in your functional abilities, such as walking, climbing up and down stairs, and sitting to stand, that you experienced following the 12 sessions of treatment?
3. Activities of daily living perception: Did you notice any significant changes in your independence and abilities related to daily activities like dressing, bathing, toileting, cooking, or going shopping following the 12 sessions of treatment?
4. Psychological aspect 1: emotions: How have your emotions and feelings related to KOA symptoms changed throughout the 12 treatment sessions?
5. Psychological aspect 2: stress and anxiety: How have your levels of stress and anxiety related to KOA symptoms changed throughout the 12 treatment sessions?
6. Psychological aspect 3: overall mood, outlook on life, and sleep-related: How have your overall mood, outlook on life, and sleep-related experiences changed throughout the 12 treatment sessions in relation to KOA symptoms?
7. Psychological aspect 4: social life: Have you found it easier or more enjoyable to socialize with others as a result of the 12 treatment sessions in relation to KOA symptoms?
8. Treatment Experience - Safety and Adverse Events: How satisfied are you with the overall safety and experience of participating in the 12 sessions of treatment, and do you have any concerns or experience any adverse effects during the process?

3.3.2 Data Analysis

The qualitative data obtained through one-on-one interviews will undergo a thematic analysis process facilitated by ATLAS.ti software programs. Thematic analysis is employed to gain an in-depth comprehension of participants' perspectives and experiences, thereby offering valuable insights to the study. This analysis encompasses several key stages, beginning with the transcription of

interview recordings into textual form. Subsequently, the transcribed data is imported into the ATLAS.ti software for thorough examination. The process includes data familiarization, which involves reviewing the interview transcripts to become well-acquainted with their content. Codes are then developed to categorize various segments of the data, and patterns and themes are identified from the coded data. To ensure the accuracy and consistency of these themes, they are refined and validated through comparison across different interviews, ensuring they faithfully represent participants' viewpoints.

3.4 Safety and ethical consideration

Safety and ethical considerations for this study will be addressed through obtaining ethical approval from the Ethics Committee of the Secretariat of Research Ethics, Universiti Kebangsaan Malaysia. Participation in this clinical trial will be voluntary, with eligible participants provided ample details about the study protocol and required to sign an informed consent form prior to participation. They will retain the option to withdraw at any time without impacting their regular treatment. Safety will be a paramount concern, with the following measures:

- Assurance of Acupuncture Safety: A wealth of studies has indicated the overall safety of acupuncture, with minimal documented risks (Bäumler et al., 2021; Chan et al., 2017; Khan et al., 2018; Lu et al., 2008; Tian et al., 2022; Vickers et al., 2012; Xu et al., 2023; Ye et al., 2023).
- Deployment of Qualified Practitioners: Skilled acupuncturists and therapists will be engaged to ensure the safe and proficient delivery of treatments.
- Implementation of Infection Control Measures: Rigorous infection control protocols will be enforced to uphold a sterile environment during acupuncture sessions. These protocols encompass hand hygiene, use of disposable needles, skin preparation, maintenance of a clean environment, proper needle handling, glove usage, and patient screening.
- Monitoring and Reporting of Adverse Effects: Adverse effects will be meticulously monitored and reported during each follow-up session to guarantee participant safety.
- Study Termination Protocol: In the event of any severe adverse effects, the study will be promptly terminated to safeguard the well-being of participants.

3.5 Funding

This study has no specific funding or grants..Participants in the study will only be responsible for paying their normal treatment fees, as outlined by their healthcare provider. Any costs specifically related to participation in the research, such as additional assessments or interventions, will be covered by Grand Care Rehab. This approach ensures that participants are not burdened with extra financial expenses, while also facilitating their involvement in the study without financial barriers.

3.6 Gantt Chart

Activity	Year 1 2024												Year 2 2025											
	1	2	3	4	5	6	7	8	9	10	11	12	1	2	3	4	5	6	7	8	9	10	11	12
Ethics application and trial protocol registration	■	■	■	■																				
Research materials and equipment procurement: Acugraph system		■	■	■																				
Screening, recruitment, randomization & intervention				■	■	■	■	■	■	■	■	■	■											
Data collection (short-term effects)-Phase 1								■	■	■	■	■	■											
Data collection (follow-up assessment) - Phase 2										■	■	■	■	■	■	■	■							
Data collection - Phase 3														■	■	■	■	■						
Data analysis												■	■	■	■	■	■	■	■	■				
Reporting the findings and journal article publication																■	■	■	■	■	■	■	■	■

4.0 References

AHMAD, M. A., SINGH, A., KAUR, D., QING, C. W., ABD RAHMAN, N. N. A., HENDRI, M., & NOOR, E. (2018). Knee Osteoarthritis and Its Related Issues: Patients' Perspective. *Malaysian Journal of Health Sciences/Jurnal Sains Kesihatan Malaysia*, *16*(2).

Alghadir, A. H., & Khan, M. (2022). Factors affecting pain and physical functions in patients with knee osteoarthritis: An observational study. *Medicine*, *101*(47).

Allen, K. D., Bongiorni, D., Caves, K., Coffman, C. J., Floegel, T. A., Greysen, H. M., Hall, K. S., Heiderscheit, B., Hoenig, H. M., & Huffman, K. M. (2019). STepped exercise program for patients with knee OsteoArthritis (STEP-KOA): protocol for a randomized controlled trial. *BMC musculoskeletal disorders*, *20*(1), 1-13.

Atalay, S. G., Durmus, A., & Gezginaslan, Ö. (2021). The effect of acupuncture and physiotherapy on patients with knee osteoarthritis: a randomized controlled study. *Pain Physician*, *24*(3), E269.

Bannuru, R. R., Osani, M., Vaysbrot, E., Arden, N., Bennell, K., Bierma-Zeinstra, S., Kraus, V., Lohmander, L. S., Abbott, J., & Bhandari, M. (2019). OARSI guidelines for the non-surgical management of knee, hip, and polyarticular osteoarthritis. *Osteoarthritis and cartilage*, *27*(11), 1578-1589.

Bäumler, P., Zhang, W., Stübinger, T., & Irnich, D. (2021). Acupuncture-related adverse events: systematic review and meta-analyses of prospective clinical studies. *BMJ open*, *11*(9), e045961.

Chan, M. W., Wu, X. Y., Wu, J. C., Wong, S. Y., & Chung, V. C. (2017). Safety of acupuncture: overview of systematic reviews. *Scientific reports*, *7*(1), 3369.

Chang, T.-F., Liou, T.-H., Chen, C.-H., Huang, Y.-C., & Chang, K.-H. (2012). Effects of elastic-band exercise on lower-extremity function among female patients with osteoarthritis of the knee. *Disability and rehabilitation*, *34*(20), 1727-1735.

Chao, J., Jing, Z., Xuehua, B., Peilei, Y., & Qi, G. (2021). Effect of systematic exercise rehabilitation on patients with knee osteoarthritis: a randomized controlled trial. *Cartilage*, *13*(1_suppl), 1734S-1740S.

Chen, A., Gupte, C., Akhtar, K., Smith, P., & Cobb, J. (2012). The global economic cost of osteoarthritis: how the UK compares. *Arthritis*, *2012*.

Cooper, C., Rannou, F., Richette, P., Bruyère, O., Al-Daghri, N., Altman, R. D., Brandi, M. L., Collaud Basset, S., Herrero-Beaumont, G., & Migliore, A. (2017). Use of intraarticular hyaluronic acid in the management of knee osteoarthritis in clinical practice. *Arthritis care & research*, *69*(9), 1287-1296.

Cui, A., Li, H., Wang, D., Zhong, J., Chen, Y., & Lu, H. (2020). Global, regional prevalence, incidence and risk factors of knee osteoarthritis in population-based studies. *EClinicalMedicine*, *29*.

Dewey, C. W., & Xie, H. (2021). The scientific basis of acupuncture for veterinary pain management: A review based on relevant literature from the last two decades. *Open Veterinary Journal*, *11*(2), 203-209.

Ding, N., Li, L., Song, K., Huang, A., & Zhang, H. (2020). Efficacy and safety of acupuncture in treating post-traumatic stress disorder: A protocol for systematic review and meta-analysis. *Medicine*, *99*(26).

Fernandes, L., Hagen, K. B., Bijlsma, J. W., Andreassen, O., Christensen, P., Conaghan, P. G., Doherty, M., Geenen, R., Hammond, A., & Kjeken, I. (2013). EULAR recommendations for the non-pharmacological core management of hip and knee osteoarthritis. *Annals of the rheumatic diseases*, *72*(7), 1125-1135.

Focht, B. C. (2006). Effectiveness of exercise interventions in reducing pain symptoms among older adults with knee osteoarthritis: a review. *Journal of aging and physical activity*, *14*(2), 212-235.

Foster, N. E., Nicholls, E., Holden, M. A., Healey, E. L., Hay, E. M., & team, B. t. (2023). Improving the Effectiveness of Exercise Therapy for Adults With Knee Osteoarthritis: A Pragmatic Randomized Controlled Trial (BEEP Trial). *Archives of Rehabilitation Research and Clinical Translation*, *5*(2), 100266.

Foster, N. E., Thomas, E., Barlas, P., Hill, J. C., Young, J., Mason, E., & Hay, E. M. (2007). Acupuncture as an adjunct to exercise based physiotherapy for osteoarthritis of the knee: randomised controlled trial. *Bmj*, *335*(7617), 436.

Garin, N., Koyanagi, A., Chatterji, S., Tyrovolas, S., Olaya, B., Leonardi, M., Lara, E., Koskinen, S., Tobiasz-Adamczyk, B., & Ayuso-Mateos, J. L. (2016). Global multimorbidity patterns: a cross-sectional, population-based, multi-country study. *Journals of Gerontology Series A: Biomedical Sciences and Medical Sciences*, *71*(2), 205-214.

Gill, P., & Baillie, J. (2018). Interviews and focus groups in qualitative research: an update for the digital age. *British dental journal*, *225*(7), 668-672.

Gill, P., Stewart, K., Treasure, E., & Chadwick, B. (2008). Methods of data collection in qualitative research: interviews and focus groups. *British dental journal*, *204*(6), 291-295.

Han, J., Zeng, Z., Pei, F., & Zheng, T. (2021). An implementation study of periarticular knee osteotomy in the treatment of knee osteoarthritis. *American Journal of Translational Research*, *13*(5), 4771.

Hawker, G. A. (2019). Osteoarthritis is a serious disease. *Clin Exp Rheumatol*, *37*(Suppl 120), 3-6.

Hinman, R. S., McCrory, P., Pirotta, M., Relf, I., Forbes, A., Crossley, K. M., Williamson, E., Kyriakides, M., Novy, K., & Metcalf, B. R. (2014). Acupuncture for chronic knee pain: a randomized clinical trial. *Jama*, *312*(13), 1313-1322.

Holden, M. A., Hattle, M., Runhaar, J., Riley, R. D., Healey, E. L., Quicke, J., van der Windt, D. A., Dziedzic, K., van Middelkoop, M., & Burke, D. (2023). Moderators of the effect of therapeutic exercise for knee and hip osteoarthritis: a systematic review and individual participant data meta-analysis. *The Lancet Rheumatology*.

Jia, P., Liu, J., Li, L., Luo, Y., Li, Y., Zhao, L., Liang, F., Liu, Z., Zou, K., & Tang, L. (2020). Acupuncture for knee osteoarthritis with sensitized acupoints: results from a pilot, feasibility randomized controlled trial. *Pilot and feasibility studies*, *6*(1), 1-15.

Jiang, T., Zhang, Q., Yuan, F., Zhang, F., & Guo, J. (2022). Efficacy of acupuncture and its influence on the emotional network in adult insomnia patients: protocol for a randomized controlled clinical trial. *Trials*, *23*(1), 1-11.

Johnson, A. J., Laffitte Nodarse, C., Peraza, J. A., Valdes-Hernandez, P. A., Montesino-Goicolea, S., Huo, Z., Fillingim, R. B., & Cruz-Almeida, Y. (2021). Psychological profiles in adults with knee OA-related pain: a replication study. *Therapeutic Advances in Musculoskeletal Disease*, *13*, 1759720X211059614.

Kamsan, S. S., Singh, D. K. A., Tan, M. P., & Kumar, S. (2020). The knowledge and self-management educational needs of older adults with knee osteoarthritis: A qualitative study. *PloS one*, *15*(3), e0230318.

Kaspiris, A., Hadjimichael, A. C., Lianou, I., Iliopoulos, I. D., Ntourantonis, D., Melissaridou, D., Savvidou, O. D., Papadimitriou, E., & Chronopoulos, E. (2023). Subchondral Bone Cyst Development in Osteoarthritis: From Pathophysiology to Bone Microarchitecture Changes and Clinical Implementations. *Journal of Clinical Medicine*, *12*(3), 815.

Khan, M., Adili, A., Winemaker, M., & Bhandari, M. (2018). Management of osteoarthritis of the knee in younger patients. *Cmaj*, *190*(3), E72-E79.

Kim, H., Mawla, I., Lee, J., Gerber, J., Walker, K., Kim, J., Ortiz, A., Chan, S.-T., Loggia, M. L., & Wasan, A. D. (2020). Reduced tactile acuity in chronic low back pain is linked with structural neuroplasticity in primary somatosensory cortex and is modulated by acupuncture therapy. *Neuroimage*, *217*, 116899.

Kolasinski, S. L., Neogi, T., Hochberg, M. C., Oatis, C., Guyatt, G., Block, J., Callahan, L., Copenhaver, C., Dodge, C., & Felson, D. (2020). 2019 American College of Rheumatology/Arthritis Foundation guideline for the management of osteoarthritis of the hand, hip, and knee. *Arthritis & rheumatology*, *72*(2), 220-233.

Kwak, S. G., Kwon, J. B., Seo, Y. W., & Choi, W.-K. (2023). The effectiveness of acupuncture as an adjunctive therapy to oral pharmacological medication in patient with knee osteoarthritis: A systematic review and meta-analysis. *Medicine*, *102*(11).

Langevin, H. M., & Wayne, P. M. (2018). What is the point? The problem with acupuncture research that no one wants to talk about. *The Journal of Alternative and Complementary Medicine*, *24*(3), 200-207.

Leifer, V., Katz, J., & Losina, E. (2022). The burden of OA-health services and economics. *Osteoarthritis and cartilage*, *30*(1), 10-16.

Lele, Z., Haixin, Y., Lei, Z., Jian, L., & Hongmei, L. (2019). Effect of acupuncture therapies combined with usual medical care on knee osteoarthritis. *Journal of Traditional Chinese Medicine*, *39*(01), 103.

León-Ballesteros, S., Espinosa-Morales, R., Clark-Peralta, P., Gómez-Pineda, A. G., & Guadarrama-Becerril, J. H. (2020). Kinesiotape and quadriceps strengthening with elastic band in women with knee osteoarthritis and overweight or obesity. A randomized clinical trial. *Reumatología Clínica (English Edition)*, *16*(1), 11-16.

Li, J., Li, Y.-X., Luo, L.-J., Ye, J., Zhong, D.-L., Xiao, Q.-W., Zheng, H., Geng, C.-M., Jin, R.-J., & Liang, F.-R. (2019). The effectiveness and safety of acupuncture for knee osteoarthritis: an overview of systematic reviews. *Medicine*, *98*(28).

Li, P., Zhang, Y., Li, F., Cai, F., Xiao, B., & Yang, H. (2023). The Efficacy of Electroacupuncture in the Treatment of Knee Osteoarthritis: A Systematic Review and Meta-Analysis. *Advanced Biology*, 2200304.

Li, R., Chen, L., Lin, X., Zhen, R., Xu, Y., Huang, J., & Ren, Y. (2023). Efficacy and safety of acupuncture for pregnancy-related low back pain: A systematic review and meta-analysis. *Heliyon*.

Li, R., Sun, J., Hu, H., Zhang, Q., Sun, R., Zhou, S., Zhang, H., & Fang, J. (2020). Research trends of acupuncture therapy on knee osteoarthritis from 2010 to 2019: a bibliometric analysis. *Journal of pain research*, 1901-1913.

Li, R., Zhang, P., Hu, Z., Yi, Y., Chen, L., Zhang, H., Li, H., Liu, W., & Wu, M. (2020). Efficacy and safety of pricking-blood therapy for acute gouty arthritis: A protocol for systematic review and meta-analysis. *Medicine (Baltimore)*, *99*(50), e23521. https://doi.org/10.1097/MD.0000000000023521

Li, Y.-X., Xiong, J., Zhang, Z., Liao, K., Zhou, X.-H., Li, J., Xiang, J., & Xu, L.-L. (2023). Research Trends of the Research and Development of Acupuncture and Moxibustion Therapy on Lumbar Disc Herniation: A Bibliometric Analysis. *Journal of pain research*, 1835-1853.

Lim, T.-K., Ma, Y., Berger, F., & Litscher, G. (2018). Acupuncture and neural mechanism in the management of low back pain—an update. *Medicines*, *5*(3), 63.

Lin, L.-L., Li, H.-P., Yang, J.-W., Hao, X.-W., Yan, S.-Y., Wang, L.-Q., Yu, F.-T., Shi, G.-X., & Liu, C.-Z. (2021). Acupuncture for psychological disorders caused by chronic pain: a review and future directions. *Frontiers in Neuroscience*, *14*, 626497.

Lin, X., Huang, K., Zhu, G., Huang, Z., Qin, A., & Fan, S. (2016). The effects of acupuncture on chronic knee pain due to osteoarthritis: a meta-analysis. *JBJS*, *98*(18), 1578-1585.

Liu, C.-Y., Tu, J.-F., Lee, M. S., Qi, L.-Y., Yu, F.-T., Yan, S.-Y., Li, J.-L., Lin, L.-L., Hao, X.-W., & Su, X.-T. (2022). Is acupuncture effective for knee osteoarthritis? A protocol for a systematic review and meta-analysis. *BMJ open*, *12*(1), e052270.

Liu, J., Li, Y., Li, L., Luo, X., Li, N., Yang, X., Zhang, H., Liu, Z., Kang, D., & Luo, Y. (2022). Effects of acupuncture at acupoints with lower versus higher pain threshold for knee osteoarthritis: a multicenter randomized controlled trial. *Chinese Medicine*, *17*(1), 1-12.

Lu, W., Dean-Clower, E., Doherty-Gilman, A., & Rosenthal, D. S. (2008). The value of acupuncture in cancer care. *Hematology/oncology clinics of North America*, *22*(4), 631-648.

MacPherson, H., & Hammerschlag, R. (2012). Acupuncture and the emerging evidence base: contrived controversy and rational debate. *Journal of Acupuncture and Meridian Studies*, *5*(4), 141-147.

Magni, A., Agostoni, P., Bonezzi, C., Massazza, G., Menè, P., Savarino, V., & Fornasari, D. (2021). Management of osteoarthritis: expert opinion on NSAIDs. *Pain and Therapy*, 1-26.

Mamipour, H., Negahban, H., Aval, S. B., Zaferanieh, M., Moradi, A., & Kachooei, A. R. (2023). Effectiveness of physiotherapy plus acupuncture compared with physiotherapy alone on pain, disability and grip strength in people with carpal tunnel syndrome: A randomized clinical trial. *Journal of Bodywork and Movement Therapies*, *35*, 378-384.

Mat, S., Jaafar, M. H., Ng, C. T., Sockalingam, S., Raja, J., Kamaruzzaman, S. B., Chin, A.-V., Abbas, A. A., Chan, C. K., & Hairi, N. N. (2019). Ethnic differences in the prevalence, socioeconomic and health related risk factors of knee pain and osteoarthritis symptoms in older Malaysians. *PloS one*, *14*(11), e0225075.

Mi, B.-H., Wang, X.-Z., Yang, J.-W., Shi, G.-X., Zhang, W.-Z., Jin, L.-N., Yang, L.-S., Liu, D.-H., Kang, S.-B., & Zhou, H. (2023). Thermographic evaluation of acupoints in lower limb region of individuals with osteoarthritis: A cross-sectional case-control study protocol. *PloS one*, *18*(4), e0284381.

Miao, C., Li, X., & Zhang, Y. (2023). Effect of acupuncture on BDNF signaling pathways in several nervous system diseases. *Frontiers in Neurology*, *14*.

Mist, S. D., Aickin, M., Kalnins, P., Cleaver, J., Batchelor, R., Thorne, T., Chamberlin, S., Tippens, K., & Colbert, A. P. (2011). Reliability of AcuGraph system for measuring skin conductance at acupoints. *Acupuncture in Medicine*, *29*(3), 221-226.

Mobasheri, A., Saarakkala, S., Finnilä, M., Karsdal, M. A., Bay-Jensen, A.-C., & van Spil, W. E. (2019). Recent advances in understanding the phenotypes of osteoarthritis. *F1000Research*, *8*.

Nguyen, T. V. (2014). Osteoarthritis in southeast Asia. *International Journal of Clinical Rheumatology*.

Pazit, L., Jeremy, D., Nancy, B., Michael, B., George, E., & Hill, K. D. (2018). Safety and feasibility of high speed resistance training with and without balance exercises for knee osteoarthritis: a pilot randomised controlled trial. *Physical Therapy in Sport*, *34,* 154-163.

Primorac, D., Molnar, V., Rod, E., Jeleč, Ž., Čukelj, F., Matišić, V., Vrdoljak, T., Hudetz, D., Hajsok, H., & Borić, I. (2020). Knee osteoarthritis: a review of pathogenesis and state-of-the-art non-operative therapeutic considerations. *Genes*, *11*(8), 854.

Rathbun, A. M., Stuart, E. A., Shardell, M., Yau, M. S., Baumgarten, M., & Hochberg, M. C. (2018). Dynamic effects of depressive symptoms on osteoarthritis knee pain. *Arthritis care & research*, *70*(1), 80-88.

Rees, M.-K. L. (2017). *Evaluation of the effectiveness of laser acupuncture on osteo-arthritic knee pain: a randomised, double-blind, placebo-controlled clinical research trial*

Richebé, P., Capdevila, X., & Rivat, C. (2018). Persistent postsurgical pain: pathophysiology and preventative pharmacologic considerations. *Anesthesiology, 129*(3), 590-607.

Rizvi, M. R., Sharma, A., Hasan, S., Ahmad, F., Asad, M. R., Iqbal, A., & Alghadir, A. H. (2023). Exploring the impact of integrated polyvagal exercises and knee reinforcement in females with grade II knee osteoarthritis: a randomized controlled trial. *Scientific reports*, *13*(1), 18964.

Tang, L., Jia, P., Zhao, L., Kang, D., Luo, Y., Liu, J., Li, L., Zheng, H., Li, Y., & Li, N. (2018). Acupuncture treatment for knee osteoarthritis with sensitive points: protocol for a multicentre randomised controlled trial. *BMJ open*, *8*(10), e023838.

Testa, G., Giardina, S. M. C., Culmone, A., Vescio, A., Turchetta, M., Cannavò, S., & Pavone, V. (2021). Intra-articular injections in knee osteoarthritis: A review of literature. *Journal of functional morphology and kinesiology*, *6*(1), 15.

Thompson, S. (2017). The Difference between Acupuncture and Dry Needling a Historical and Practical Application. *International Journal of Complementary and Alternative Medicine*, *10*.

Tian, H., Huang, L., Sun, M., Xu, G., He, J., Zhou, Z., Huang, F., Liu, Y., & Liang, F. (2022). Acupuncture for knee osteoarthritis: a systematic review of randomized clinical trials with meta-analyses and trial sequential analyses. *BioMed Research International*, *2022*.

Vickers, A. J., Cronin, A. M., Maschino, A. C., Lewith, G., MacPherson, H., Foster, N. E., Sherman, K. J., Witt, C. M., Linde, K., & Acupuncture Trialists' Collaboration, f. t. (2012). Acupuncture for chronic pain: individual patient data meta-analysis. *Archives of internal medicine*, *172*(19), 1444-1453.

Wang, L., Chen, H., Lu, H., Wang, Y., Liu, C., Dong, X., Chen, J., Liu, N., Yu, F., & Wan, Q. (2020). The effect of transtheoretical model-lead intervention for knee osteoarthritis in older adults: a cluster randomized trial. *Arthritis Research & Therapy*, *22*(1), 1-14.

Wang, L., Xian, J., Sun, M., Wang, X., Zang, X., Zhang, X., Yu, H., & Tan, Q.-W. (2022). Acupuncture for emotional symptoms in patients with functional gastrointestinal disorders: A systematic review and meta-analysis. *PloS one*, *17*(1), e0263166.

Wang, Z., Zhai, F., Zhao, X., Zhao, G., Li, N., Zhang, F., & Liu, J. (2020). The efficacy and safety of acupuncture for perimenopausal insomnia: A protocol for a network meta-analysis. *Medicine (Baltimore)*, *99*(52), e23741. https://doi.org/10.1097/MD.0000000000023741

White, A., Hayhoe, S., Hart, A., & Ernst, E. (2001). Adverse events following acupuncture: prospective survey of 32 000 consultations with doctors and physiotherapists. *Bmj*, *323*(7311), 485-486.

Williamson, L., Wyatt, M., Yein, K., & Melton, J. (2007). Severe knee osteoarthritis: a randomized controlled trial of acupuncture, physiotherapy (supervised exercise) and standard management for patients awaiting knee replacement. *Rheumatology*, *46*(9), 1445-1449.

Wu, N., Huang, J., Yang, X., Guo, J., Liu, F., Gu, Y., Liu, Y., Zhang, Z., Yin, S., & Feng, X. (2020). The long-term effect of acupuncture for patients with knee osteoarthritis: Study protocol for a randomized controlled trial. *Medicine, 99*(42).

Xie, F., Kovic, B., Jin, X., He, X., Wang, M., & Silvestre, C. (2016). Economic and humanistic burden of osteoarthritis: a systematic review of large sample studies. *Pharmacoeconomics*, *34*, 1087-1100.

Xu, X., Zheng, Y., Jiang, M., Hu, H., He, X., Fang, J., & Jiang, Y. (2023). Research Trends on Acupuncture for Shoulder Pain Treatment Over the Past 15 Years: A Bibliometric Analysis. *Journal of pain research*, 2433-2446.

Yao, Q., Wu, X., Tao, C., Gong, W., Chen, M., Qu, M., Zhong, Y., He, T., Chen, S., & Xiao, G. (2023). Osteoarthritis: pathogenic signaling pathways and therapeutic targets. *Signal Transduction and Targeted Therapy*, *8*(1), 56.

Ye, Y., Li, S., Xiao, L., & Chang, J. (2023). Complementary therapies for neurological disorders: from bench to clinical practices. *Frontiers in Neurology*, *14*, 1224144.

Zamri, N., Harith, S., Mat-Hassan, N., & Ong, Y. (2021). Nutritional status and health-related quality of life among knee and hip osteoarthritis patients under rehabilitation care in Kuala Nerus, Terengganu, Malaysia. *Malaysian orthopaedic journal*, *15*(2), 77. https://www.ncbi.nlm.nih.gov/pmc/articles/PMC8381666/pdf/moj-15-077.pdf

Zeng, C.-Y., Zhang, Z.-R., Tang, Z.-M., & Hua, F.-Z. (2021). Benefits and mechanisms of exercise training for knee osteoarthritis. *Frontiers in physiology*, *12*, 2267.

Zhang, Q., Fang, J., Chen, L., Wu, J., Ni, J., Liu, F., & Sun, J. (2020). Different kinds of acupuncture treatments for knee osteoarthritis: a multicentre, randomized controlled trial. *Trials*, *21*, 1-10.

Zhang, Q., Zhou, M., Huo, M., Si, Y., Zhang, Y., Fang, Y., & Zhang, D. (2023). Mechanisms of acupuncture–electroacupuncture on inflammatory pain. *Molecular Pain*, *19*, 17448069231202882.

Zhang, Y., & Wang, C. (2020). Acupuncture and chronic musculoskeletal pain. *Current rheumatology reports*, *22*, 1-11.

Zhang, Y., Wang, Z., Du, J., Liu, J., Xu, T., Wang, X., Sun, M., Wen, Y., Li, D., & Liao, H. (2021). Regulatory effects of acupuncture on emotional disorders in patients with menstrual migraine without aura: a resting-state fMRI study. *Frontiers in Neuroscience*, *15*, 726505.

Zhang, Z., & Zhu, M. (2023). Holistic View of TCM on Cancer Integrative Therapy. *Future Integrative Medicine*, *2*(3), 159-167.

Zhou, D., Xia, X., Ling, J., & Liu, C. (2023). Regulating spleen and stomach can improve bone and joint function of knee osteoarthritis patients complicated with osteoporosis. *American Journal of Translational Research*, *15*(9), 5769.

Zhou, K., Fan, A. Y., & Wang, T. (2016). Acupuncture for Chronic Knee Pain: A Critical Appraisal of an Australian Randomized Controlled Trial. *Medical Acupuncture*, *28*(1), 40-45.

PARTICIPANT INFORMATION SHEET

Research Title:

Comparing the effects of computerized versus manual methods of identifying point-specific acupuncture as an adjunct to physiotherapy in the management of knee osteoarthritis: A randomized controlled trial

Introduction:

In recent years, there has been a growing concern regarding the intervention on knee arthritis (KOA) patients. The effectiveness of physiotherapeutic exercise and acupuncture has been proven worldwide, however the implementation, effectiveness on KOA patients remain insufficiently explored in term of combination of both treatment and selection of acupoints remain unstandardized.

Purpose of Study:

This study aims to fill these gaps by investigating the effects of a computerized method for identifying point-specific acupuncture generated by the Acugraph system when combined with physiotherapy on knee pain, physical function, psychological well-being, and health perception. Additionally, the research seeks to examine the correlation between improvements in KOA physical function, assessed through established tools like KOOS and TUG, and the AcuGraph Personal Integrated Energetics (PIE) score which serve as an indicator of the overall health of the meridian energy system.

What will the study involve?

If you are willing to participate, you would be allocated into one of the following groups:

a) Group 1 (25 participants), Comp_AcuPhysio: computer selected acupoints and physiotherapeutic exercise
b) Group 2 (25 participants), Man_AcuPhysio: manually selected acupoints and physiotherapeutic exercise

Treatments will be conducted once a week for a consecutive 12 weeks. Several assessments (physical test and questionnaires) will be carried out during the study at two intervals; before treatment (week-0) and post-treatment (week-12). The list of outcomes assessments will be as follows:

1. Knee Injury and Osteoarthritis Outcome Score (KOOS):

- Description: A standardized questionnaire assessing knee function and symptoms related to knee osteoarthritis.
- Purpose: To evaluate the impact of knee osteoarthritis on various aspects of daily life and function.
- Duration: The time to complete the KOOS questionnaire takes around 10 minutes.

2. Active knee flexion range of motion (ROM):
- Description: Measurement of the maximum range of motion achievable by actively bending the knee joint by clinician.
- Purpose: To assess the flexibility and mobility of the knee joint.
- Duration: The test usually takes 3 minutes to perform.

3. The Timed Up and Go Test
- Description: A functional mobility test that measures the time taken for an individual to stand up from a chair, walk a short distance, turn around, walk back, and sit down again.
- Purpose: To assess balance, mobility, and the risk of falls.
- Duration: The test typically takes less than 3 minutes to complete.

4. The WHO-5 Well-Being Index & Short Form 36 Health Survey (SF-36):

- Description: Standardized questionnaires assessing psychological well-being and overall health-related quality of life.
- Purpose: To evaluate mental health, emotional well-being, and general physical health status.
- Duration: The time to complete both questionnaires takes around 5 minutes for each.

5. Acugraph System (PIE Score) Evaluation
- Description: Assessment of meridian energy balance and overall health using the Acugraph system.

- Purpose: To measure and analyze the energy flow through acupuncture meridians and identify imbalances.
- Duration: The evaluation process with the Acugraph system usually takes around 10 minutes.

Benefits:

Your participation could help in the foundation for evidence-based recommendations. Our study explores the relationship between improved knee function in KOA, as measured by tools like the AcuGraph Personal Integrated Energetics (PIE) score. This not only deepens our understanding of individual health but also empowers patients to play an active role in making decisions about their care. If we prove that combining acupuncture and physiotherapy effectively treats knee osteoarthritis, it has the potential to be a recommended alternative approach.

Risks

Possible risks include bruises, skin hematoma, redness and mild need pin prick pain. In order to minimize the risks, we emphasize on these measures:

1.Qualified Practitioners: by recruiting qualified and licensed acupuncturists who adhere to safety standards and guidelines. This helps minimize the risk of errors in needle insertion and manipulation.

2.Adverse Event Monitoring: Implement documenting and reporting adverse events throughout the trial. This includes any unexpected, undesirable, or harmful effects related to acupuncture treatment.

3.Training in Safety Procedures: Ensure that practitioners are trained in safety procedures and emergency response protocols to address any adverse events promptly.

4.Needle Hygiene and Sterilization: Emphasize proper needle hygiene and sterilization to prevent infections or other complications associated with contaminated needles.

Do you have to take part?

Participation in this study is voluntary. If you agree to take part, then you will be asked to sign the “Informed Consent Form”. You will be given a copy of the form and this Information Sheet. Should you decide to participate, you can still withdraw from the study

without penalty. Your data will not be used and will be discarded. The researcher may also remove you from the study for a variety of reasons. In this event, you will not be penalized.

Data & Confidentiality:

Information extracted from the study will be interpreted with statistical analysis for testing purposes. The information will be presented as a whole without any specific individuals being mentioned. Your information will be kept private and will not be disclosed to any third party. The data from this study will be made into a report which may be published. Access to the data is only by the research team. The data will be reported in a collective manner with no reference to an individual. Hence your identity will be kept confidential.

Payment and compensation:

You will not be paid to participate in this study.

Who can I ask about the study?

If you have any questions, you can direct them to the research team.

Dr. Mohd Azzuan Bin Ahmad,
University Lecturer,
Physiotherapy Programme, Centre for Rehabilitation and Special Needs (iCaReHab),
Faculty of Health Sciences, Universiti Kebangsaan Malaysia
Jalan Raja Muda Abdul Aziz, 50300 Kuala Lumpur
Malaysia
Mobile: 012-3297292

Lee Chai Li
Physiotherapy Master Student,
Faculty of Health Sciences, Universiti Kebangsaan Malaysia
Jalan Raja Muda Abdul Aziz, 50300 Kuala Lumpur
Malaysia
Mobile: 016-6002435

Dr Tan Chee Hou
Co Researcher,

83,Jalan MPK 2,Medan Perniagaan Kepayang, Bukit Kepayang, 70200 Seremban, Negeri Sembilan
Mobile: 016-2798982

PARTICIPANT INFORMED CONSENT FORM

Research Title: Comparing the effects of computerized versus manual methods of identifying point-specific acupuncture as an adjunct to physiotherapy in the management of knee osteoarthritis: A randomized controlled trial

Researcher's Name: Lee Chai Li, Tan Chee Hou, Angeline Low Ann Je, Lee Zi Lin, Tay Yan Ling, Peng Yan, Mohd Azzuan Ahmad

I, ……………………………………, IC No : …………………......

- have read the information in the Participant Information Sheet **including information regarding the risk in this study.**
- have been given time to think about it and all of my questions have been answered to my satisfaction.
- understand that I may freely choose to withdraw from this study at anytime without reason and without repercussion
- understand that my anonymity will be ensured in the write-up.

I voluntarily agree to be part of this research study, to follow the study procedures, and to provide necessary information to the doctor, nurses, or other staff members, as requested.

……………………………… ……………………..
(Signature) (Date)

………………………………<br>Witness (if any)	………………………………<br>Researcher
………………………………<br>(Signature)	………………………………<br>(Signature)
………………………………<br>(IC Number)	………………………………<br>(IC Number)
……………………….…<br>(Date)	………………………………<br>(Date)
